# Short Time-Series Expression Transcriptome Data Reveal the Gene Expression Patterns of Dairy Cow Mammary Gland as Milk Yield Decreased Process

**DOI:** 10.3390/genes12060942

**Published:** 2021-06-20

**Authors:** Yongliang Fan, Ziyin Han, Xubin Lu, Abdelaziz Adam Idriss Arbab, Mudasir Nazar, Yi Yang, Zhangping Yang

**Affiliations:** 1 College of Animal Science and Technology, Yangzhou University, Yangzhou 225009, China; dx120170088@yzu.edu.cn (Y.F.); ZiyinHan@126.com (Z.H.); dx120180094@yzu.edu.cn (X.L.); arbabtor@yahoo.com (A.A.I.A.); drmudasirnazar457@gmail.com (M.N.); 2 Joint International Research Laboratory of Agriculture & Agri-Product Safety, Ministry of Education, Yangzhou University, Yangzhou 225009, China; 3 Jiangsu Co-Innovation Center for the Prevention and Control of Important Animal Infectious Diseases and Zoonoses, Yangzhou University College of Veterinary Medicine, Yangzhou 225009, China; yangyi@yzu.edu.cn

**Keywords:** mammary gland, short time-series expression miner, transcriptome, differentially expressed genes

## Abstract

The existing research on dairy cow mammary gland genes is extensive, but there have been few reports about dynamic changes in dairy cow mammary gland genes as milk yield decrease. For the first time, transcriptome analysis based on short time-series expression miner (STEM) and histological observations were performed using the Holstein dairy cow mammary gland to explore gene expression patterns in this process of decrease (at peak, mid-, and late lactation). Histological observations suggested that the number of mammary acinous cells at peak/mid-lactation was significantly higher than that at mid-/late lactation, and the lipid droplets area secreted by dairy cows was almost unaltered across the three stages of lactation (*p* > 0.05). Totals of 882 and 1439 genes were differentially expressed at mid- and late lactation, respectively, compared to peak lactation. Function analysis showed that differentially expressed genes (DEGs) were mainly related to apoptosis and energy metabolism (fold change ≥ 2 or fold change ≤ 0.5, *p*-value ≤ 0.05). Transcriptome analysis based on STEM identified 16 profiles of differential gene expression patterns, including 5 significant profiles (false discovery rate, FDR ≤ 0.05). Function analysis revealed DEGs involved in milk fat synthesis were downregulated in Profile 0 and DEGs in Profile 12 associated with protein synthesis. These findings provide a foundation for future studies on the molecular mechanisms underlying mammary gland development in dairy cows.

## 1. Introduction

Milk is a traditional food source with balanced nutrition, and it has constituted an important part of the human diet since ancient times [1]. The mammary gland, which is the sole organ of milk synthesis, secretion, and storage, determines the yield and quality of milk [2]. The mammary gland is highly plastic and undergoes repeated cycles of structural growth, development, differentiation, and regression [3]. Lactation is a complex process, which includes several physiological changes, including mammary gland development and milk synthesis and secretion [4]. The lactation process is classically divided into different stages in dairy cows, including early, mid-, and late lactation. Milk yield and composition are influenced not only by nutrition, pasture management, age, and season but also by the stage of lactation [5,6]. In a lactation period, the milk yield and composition change regularly with an increasing number of lactation days. The synthesis and secretion of bovine milk are regulated by a number of genes [7,8]. Exploring the gene expression levels of the bovine mammary gland is helpful in revealing the biological mechanism of mammary morphogenesis, metabolic activity, and milk composition synthesis [9].

In the past years, Bionaz et al. [10] used an annotated bovine oligonucleotide microarray containing >10,000 unique elements and revelated the function of the bovine mammary transcriptome during the lactation cycle. RNA sequencing has a more sensation for low abundance transcripts than microarray and has the ability to distinguish isoforms from the identification of gene variants [11]. In recent years, transcriptome sequencing has been used to analyze the transcripts of the mammary gland of Holstein dairy cows at different periods of lactation. Gao et al. [12] measured the transcripts in the mammary gland collected on the 35th day before parturition, the 7th day before parturition, and the 3rd day after parturition, and found that the mammary gland began to secrete milk not only by a gain of function but also by a broad suppression of function to effectively push most of the cells’ resources toward lactation. To explore the gene expression profile alterations during non-lactation (early stage of pregnancy) versus mid-lactation, transcriptomic analysis was used to identify differentially expressed genes (DEGs) in the mammary gland of Holstein dairy cows [7]. In research by Lin et al. [8], the mammary DEGs in the cows between early lactation and the mid-dry period were used to facilitate the investigation of the mechanisms underlying lactation and mammary gland remodeling in Holstein dairy cows. To our knowledge, transcriptome sequencing has often been used to investigate variation in gene profiles in nonlactating and lactating Holstein dairy cows [7,8]. However, the transcriptome analysis of the bovine mammary gland in the period of lactation decline has not been studied.

It is generally recognized that a relatively small number of transcription factors regulates tissue-specific or developmental stage-specific gene expression [13]. According to a reasonable assumption of Pizzi et al. [13], genes with similar expression seem to similar biological functions and are co-regulated frequently and regulated in a coordinated way possibly. Therefore, it is necessary to explore the expression pattern of genes to reveal the gene set regulating traits of mammary glands or milk of dairy cows. The short time-series expression miner (STEM) clustering method applies a large number of genes and the number of few time points to identify expression profiles with statistically significant temporal and the genes involved in these profiles [14]. STEM supports gene ontology (GO) annotation of gene sets with the same temporal expression pattern [15], offering an approach for a statistically rigorous biological interpretation of typical temporal expression patterns efficiently. STEM provides a new and feasible idea to clarify the typical temporal expression patterns in the mammary gland of dairy cows.

To reveal the expression changes of key mammary gland genes related to lactation decline in dairy cows, we, for the first time, determined the expression variations in mammary gland genes of Holstein dairy cow during peak, mid-, and late lactation, and we analyzed the data using the short time-series expression miner (STEM) clustering method. Our findings will help to clarify the mechanism of milk production and will have potential application value for improving the quality of dairy products.

## 2. Materials and Methods

### 2.1. Sample Collection

A total of 33 Holstein dairy cows were selected from the experimental farm of Yangzhou University. These dairy cows had similar body weights (619.37 ± 23.41 kg) and were in second parity. They were fed with total mixed ration (TMR) and provided with adequate water ad libitum [7]. Milk samples were collected at the 90th (peak lactation), 180th (mid-lactation), and 270th (late lactation) days postpartum for milk composition determination. At each sampling date, the milk samples were, respectively, collected from the left anterior mammary region of each dairy cow (n = 33). All the milk samples underwent a determination of somatic cell count (SCC) to exclude individuals with subclinical mastitis. The prerequisite of variance analysis is the independence of random error, normality, and homogeneity. Because the SCC does not conform to normal distribution, it cannot be applied to variance analysis, and it should be calculated to SCS for variance analysis (SCS = log_2_SCC/100 + 3) [16]. At 270, 180, and 270 d postpartum, approximately 3 g of the mammary glands of each dairy cow (A, B, C) was collected from the left anterior mammary region via a surgical method [17,18]. First, the sampling site was shaved and disinfected with 75% ethanol. A dairy cow was anesthesia using 35 mg Su-Mian-Xin (intravenous injection) as a basic general anesthesia agent and 1 mL procaine (subcutaneous injection) as local anesthesia. Next, the site was dissected using a sterile scalpel, and connective tissue was removed by blunt dissection to explore the mammary parenchyma. Then, approximately 3 g of mammary gland was collected and washed with DEPC sterile water three times. Eventually, the sample was transferred into a sterile EP tube and flash-frozen in liquid nitrogen until RNA isolation. After sample collection, the skin incision was sutured using 11 mm Michel wound clips (Henry Stein, Melville, NY, USA) and disinfected with iodine ointment (Povidone Iodine Ointment, Guangzhou, China). Around 0.5 g of mammary grand was applied for RNA isolation in each sample. Additionally, approximately 1 g of mammary tissue was used for sections.

### 2.2. Milk Composition Detection and Histological Examination

The concentrations of milk fat, protein, and lactose were determined using mid-infrared spectrometry (MilkoScan Minor, Foss Analytics, Hillerød, Denmark) [19]. Paraffin sections were stained with hematoxylin and eosin (HE) for routine histological studies, and the sections were made in the same manner as Li et al. [20]. Sections were analyzed by light microscopy using a Nikon fluorescence microscope (Nikon, Tokyo, Japan). Image-pro Plus v6.0 was used to realize area analysis of the mammary gland acinus. The two acini with the area closest to the average area were selected at 90, 180, and 270 d to calculate the average number of nuclei in each acinus. At each sampling time, we recorded the lipid droplet area of the acini that had upper quartile area, median area, or lower quartile area.

### 2.3. Transcriptome Sequencing

A total of nine samples were used for RNA isolation. Total RNA was extracted using the mirVana™ miRNA Isolation Kit (Ambion, CA, USA, catalog #1561) following the manufacturer’s protocol. The integrity of total RNAs was evaluated on an Agilent 2100 Bioanalyzer (Agilent Technologies, Santa Clara, CA, USA). The samples with an RNA Integrity Number (RIN) ≥ 7 were used in the cDNA library construction. The libraries were constructed using a TruSeq Stranded mRNA LTSample Prep Kit (Illumina, CA, USA, catalog # RS-122-2101) according to the manufacturer’s instructions.

The libraries were sequenced on the Illumina sequencing platform (HiSeqTM 2500), generating 125 bp paired-end reads. By removing the low-quality reads and reads containing poly-N, raw reads were processed using the NGS QC Toolkit to obtain clean reads [21]. Then, the clean reads were mapped to reference bovine genome UMD3.1 using Bowtie2 2.3.5.1 [22,23].

### 2.4. Gene Expression Level Analysis

The known transcripts were identified after annotation. The fragments per kilobase of exon per million fragments mapped (FPKM) method was used to calculate each gene expression level, which is the number of fragments per kilobase length from a gene in every million fragments [24]. Per-gene read counts were obtained using HtSeq-count 0.9.1 [25]. Principal component analysis (PCA) was performed using the gene expression profiles. The DESeq R package (1.18.0) (2012) functions were used to estimate size factors, and the nbinom test was used to identify DEGs [26,27]. Fold change ≥2 or ≤0.50, and *p*-value ≤ 0.05 and were set as the threshold for DEGs. DEGs identified among different periods were visualized using a Venn diagram (https://bioinfogp.cnb.csic.es/tools/venny/index.html, accessed on 10 November 2020) [28].

### 2.5. Bioinformatics Analysis of Differentially Expressed Genes

To explore the expression pattern of DEGs in the three periods, DEGs enriched in GO terms were extracted using the annotation software DAVID 6.8 (https://david.ncifcrf.gov/, accessed on 13 November 2020) [29]. The metabolic pathway analysis of DEGs was implemented by the KOBAS 3.0 online program (http://kobas.cbi.pku.edu.cn/index.php, accessed on 13 November 2020) [30]. R based on the hypergeometric distribution was determined to identify significant GO terms and Kyoto Encyclopedia of Genes and Genomes (KEGG) pathways compared with the entire genomic background [31], setting a false discovery rate (FDR) ≤ 0.05 as a threshold. The formula for calculation was as follows (1), in which M denotes the number of genes annotated in a pathway and m denotes the number of DEGs in M; N denotes the number of genes annotated in a pathway, and n denotes the number of DEGs in N.

(1) $$p=1-\overset{m-1}{\underset{i=0}{\sum }}\frac{\left(\begin{array}{c} M\\ i \end{array}\right)\left(\begin{array}{c} N-M\\ n-i \end{array}\right)}{\left(\begin{array}{c} N\\ n \end{array}\right)}$$

### 2.6. Short Time-Series Expression Miner Analysis

The STEM clustering method was applied to analyze the expression patterns of DEGs in the bovine mammary gland during the period of lactation decline [14,32]. Each gene was assigned to the closest profile using a Pearson correlation-based distance metric. A permutation-based test was used to quantify the expected number of genes that would be assigned to each profile to determine the significance level of a given transcriptome profile [33]. Profiles with an FDR ≤ 0.05 were considered significantly enriched. Then, GO enrichment and KEGG pathway analysis were applied to consider the expression pattern of DEGs in each profile.

A protein-protein interaction (PPI) network in each significant profile was computed using the String v11.0 database [34]. Subsequently, the PPI network was visualized in Cytoscape v3.7.2 to further understand and predict the biological activity of the DEGs [35].

### 2.7. Validation of Sequencing Data by qRT-PCR

The accuracy of sequencing results was confirmed by quantitative real-time PCR (qRT-PCR). qRT-PCR was achieved using the Light Cycler^®^ 480 System (Roche, Hercules, CA, USA) with TB Green^®^ Premix Ex Taq™ II (TaKaRa, catalog #RR820). A reaction was performed with three biological replicates, and each biological replicate was performed with three technical replicates. Primers were designed in Primer 5.0 and are shown in Table S1. The annealing temperature of all primers was 60 °C. Ribosomal protein S9 (*RPS9*) and *β-actin* were used as reference genes [7,36]. The reaction system included 10 μL of 2× TB Green Premix Ex Taq II (Tli RNaseH Plus), 2 μL cDNA, 0.8 μL forward primers, 0.8 μL reverse primers, and 6.4 μL ddH_2_O to a total of 20 μL. The procedures were 95 °C for 30 s, 95 °C for 5 s, and 60 °C for 20 s, for 40 cycles. The relative expression of genes was calculated by the 2^−ΔΔCt^ method.

### 2.8. Statistical Analysis

The statistical analysis was performed using GraphPad Prism 8 (GraphPad, San Diego, CA, USA) and one-way ANOVA. The steps of one-way ANOVA include the normality test, the homogeneity of variance test, and the comparison of means. The data were used for normality and lognormality test. The result of normal distribution showed that the *p*-value summary was “ns”, suggesting that the data met the criteria of one-way ANOVA. If *p*-value ≤ 0.05 for the homogeneity of variance test, Tukey test multiple comparisons is performed. Outliers were excluded using the Rout method [37]. Data are presented as the mean ± standard deviation (SD), and *p* ≤ 0.05 (*) indicates a significant difference. In addition, the Pearson correlation coefficient was evaluated for the data obtained from sequencing and qRT-PCR.

## 3. Results 

### 3.1. Daily Milk Yield, Milk Composition, and Somatic Cell Count

Whether based on the data of 33 cows (Table S2) or 3 cows (Table 1), the daily milk yield and lactose content showed a downward trend from peak to late lactation; conversely, the percentage of milk fat and protein increased. The SCC values of all the dairy cows were below the national standard of China, 500,000/mL (Table 1 and Table S2).

### 3.2. Histological Observation

**Figure 1:**
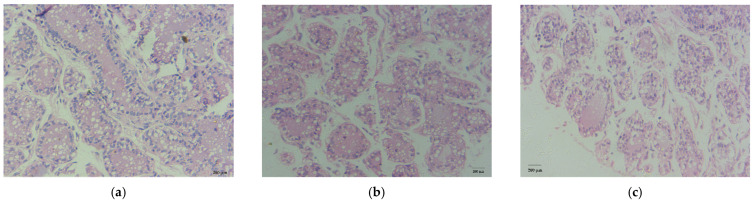
Histological sections stained with HE. (**a**) Dairy cow mammary tissue collected at 90 d of lactation. (**b**) Dairy cow mammary tissue collected at 180 d of lactation. (**c**) Dairy cow mammary tissue collected at 270 d of lactation.

The histological sections are shown in Figure 1, and descriptive summary statistics are presented in Table 2. The numbers of acini at 90, 180, and 270 d were 11, 20, and 55, respectively (File S1). The average acinus area at 90 d was about 2.44 times the average acinus area at 180 d, and the average acinus area at 180 d was about 5.46 times the average acinus area at 270 d (File S1 and Figure S1). Apparently, the nuclei number of acini decreased gradually over time of lactation (*p* < 0.05). The average area of lipid droplets was almost unaltered across the three stages of lactation (*p* > 0.05).

### 3.3. Transcriptome Sequencing Results and Quality Control

The nine complementary DNA (cDNA) libraries produced 613.44 million (M) raw reads altogether. After removal of the low-quality reads, a total of 604.64 M high-quality clean reads were obtained in the nine cDNA libraries. The sequences with Q30 ranged from 95.92% to 97.33%. The GC content reached between 47.50% and 50.00% (Table S3). These results indicate that our data were suitable for further analysis. Furthermore, the clean reads were aligned with the reference bovine genome (UMD3.1).

A principal component analysis (PCA) on the entire transcriptome data set was performed, and three clusters were generated: peak, mid-, and late lactation (Figure S2). Each cluster included genes from different dairy cows in the same stage, suggesting that the main distinctions in the mRNA expression profiles occurred in the different stages.

### 3.4. Identification of Differentially Expressed Genes

**Figure 2:**
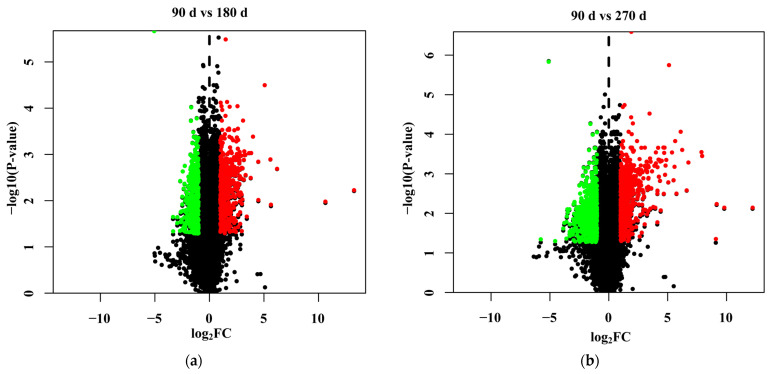
Volcano plot of differentially expressed genes (DEGs). (**a**) Volcano plot of DEGs identified for mid-lactation compared to peak lactation. (**b**) Volcano plot of DEGs identified for late lactation compared to peak lactation.

**Figure 3:**
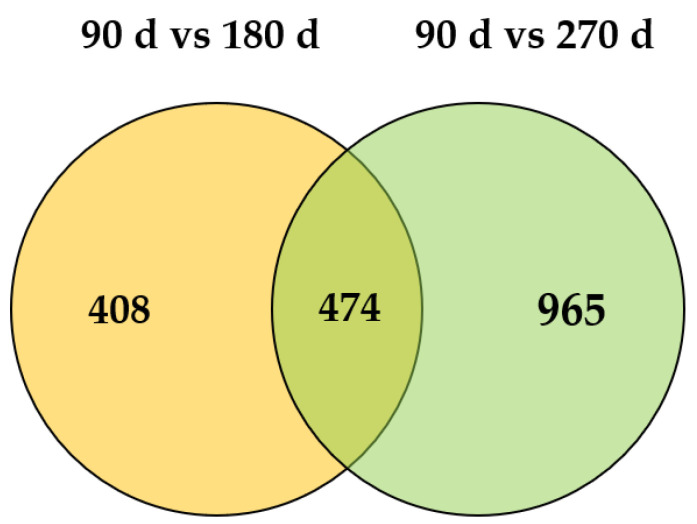
Venn diagram depicting the overlap between the two sets of differentially expressed genes.

Two comparisons of gene expression at the three time points were investigated to identify the DEGs during peak, mid-, and late lactation. More DEGs were detected with an increasing number of days of lactation. There were 882 DEGs for mid-lactation compared with peak lactation, and the expression of 1439 genes was significantly different between peak and late lactation (Figure 2). The DEG sets at the 180th and 270th days after calving were compared and are presented as a Venn diagram (Figure 3). The Venn diagram shows the overlap (474 DEGs) of the two sets of DEGs.

### 3.5. Functions of the Differentially Expressed Genes

**Figure 4:**
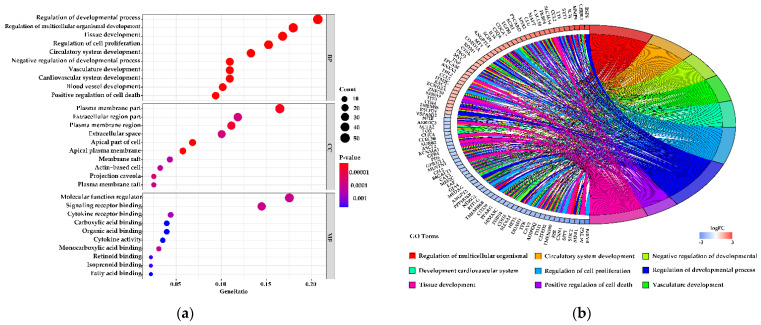

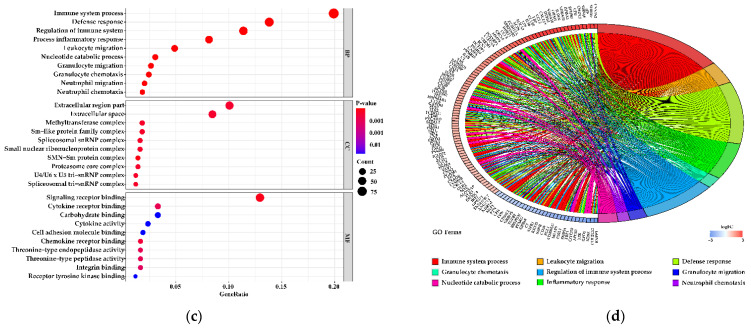
Gene ontology (GO) functional enrichment analysis of differentially expressed genes (DEGs). (**a**,**c**) Figures, respectively, show the top 10 significant GO terms for each category from (**a**) the 90 vs. 180 d group and (**c**) the 90 d vs. 270 d group. (**b**,**d**) Circos plots showing overlapping and specific responses of DEGs enriched in the nine most significant GO terms.

GO enrichment and KEGG pathway analysis were applied to assay the DEGs to further understand their biological function. The GO enrichment analysis was tested independently in three categories (GO biological process (GO-BP), GO cellular component (GO-CC), and GO molecular function (GO-MF)), and the top 10 significant terms of each category are shown in Figure 4a,c. Some of the top 20 GO-BP terms for 90 vs. 180 d were associated with tissue development (Table S4). The top 20 GO-BP terms (90 vs. 180 d) were mainly associated with cell proliferation and cell apoptosis (Table S4). Genes in the significant GO-CC terms (90 vs. 180 d) mainly involve the plasma membrane, extracellular space, cytoplasm, and apical region (Table S4). Additionally, there were some partly terms involved in functions related to the cytoplasm and apical region in the top 20 significant GO-CC terms for 90 vs. 270 d. Some of the terms from the top 20 significant GO-CC terms (90 vs. 270 d) were associated with protein complex and spliceosome (Table S5). Of the top 20 GO-MF terms for 90 vs. 270 d, 40% were related to material binding, and 15% were related to material transport (Table S5). Figure 4b,d and Figure S3 show the levels of variation in the DEGs belonging to the top 9 significant enrichment terms.

**Figure 5:**
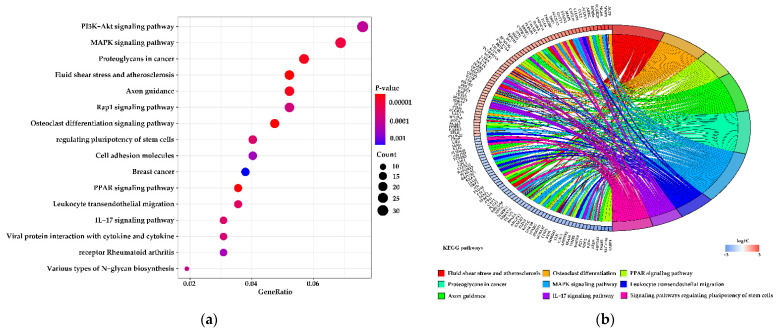

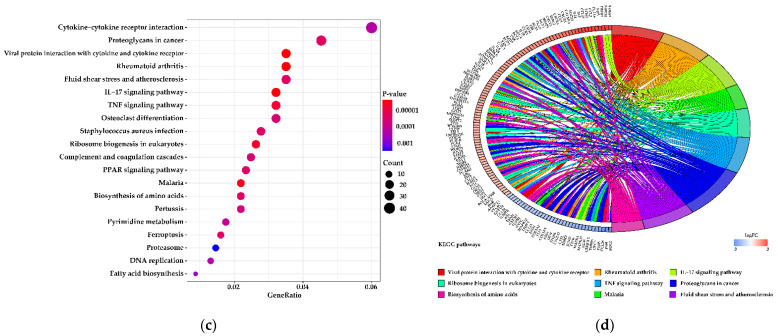
Kyoto Encyclopedia of Genes and Genomes (KEGG) pathway enrichment analysis of differentially expressed genes (DEGs). (**a**,**c**) Figures, respectively, show the top 10 significant KEGG pathways of each category from (**a**) the 90 vs. 180 d group and (**c**) the 90 vs. 270 d group. (**b**,**d**) Circos plots showing overlapping and specific responses of DEGs enriched in the top nine KEGG pathways.

Significantly enriched KEGG pathways identified between peak and mid-lactation are shown in Figure 5a, and the top 20 most abundant KEGG pathways during peak and late lactation are listed in Figure 5c. The results show that fatty acid degradation, peroxisome proliferator-activated receptor (PPAR), and mineral absorption signaling pathways were significantly different in mid- vs. peak and late vs. mid-lactation (FDR ≤ 0.05). Some of the KEGG pathways in the peak vs. mid group are related to polysaccharide biosynthesis, glycosphingolipid biosynthesis, and inhibition of cell growth. The majority of the KEGG pathways in the peak vs. late group are associated with purine and pyrimidine metabolism, DNA replication, base excision repair, and the biosynthesis and metabolism of amino acids. A single gene is usually assigned to many ontological terms due to the KEGG database structure. For this reason, the relationships between the genes and the top nine KEGG pathways were mapped with a circos plot (Figure 5b,d and Figure S4).

### 3.6. Dynamic Expression Profiles of DEGs

**Figure 6:**
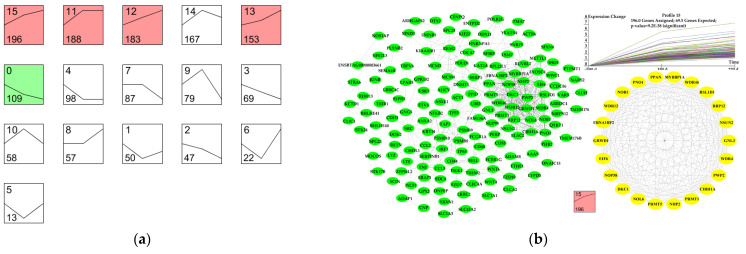

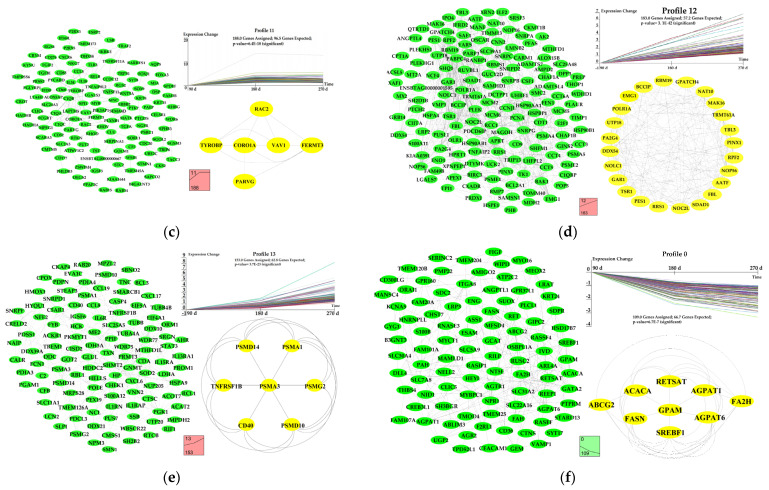
Patterns of gene expression across the three time points inferred by STEM analysis. (**a**) Sixteen candidate profiles were obtained via STEM analysis. The five colored profiles are significant profiles (FDR ≤ 0.05). (**b**–**f**) The green nodes in the PPI networks represent genes in the significant profiles. The yellow nodes in the PPI networks represent key genes in the significant profiles.

The expression profiles of 1847 DEGs (the genes in Figure 3) were determined using cluster analysis based on STEM to obtain their dynamic expression patterns in the mammary gland of Holstein dairy cow during peak, mid-, and late lactation. Sixteen candidate profiles were obtained (Figure 6), and five of them were significant (FDR ≤ 0.05, Figure 6a).

In Profile 15, there were 13 significant terms involved in negative regulation of the cell cycle, regulation of the apoptotic process, negative regulation of cell proliferation, and negative regulation of transcription (Table S6). In Profile 0, a total of nine significant GO terms and six significant KEGG pathways, including 17 genes, were associated with milk fat synthesis (Table S7). In Profile 12, the significant terms mainly participated in protein synthesis (Table S8).

The DEGs in each profile were mapped to the PPI network of cattle to construct a differentially expressed PPI network (Figure 6). The PPI network revealed the biological activity and interactive relationships of proteins encoded by DEGs.

### 3.7. Verification Transcriptome Data of qRT-PCR

**Figure 7:**
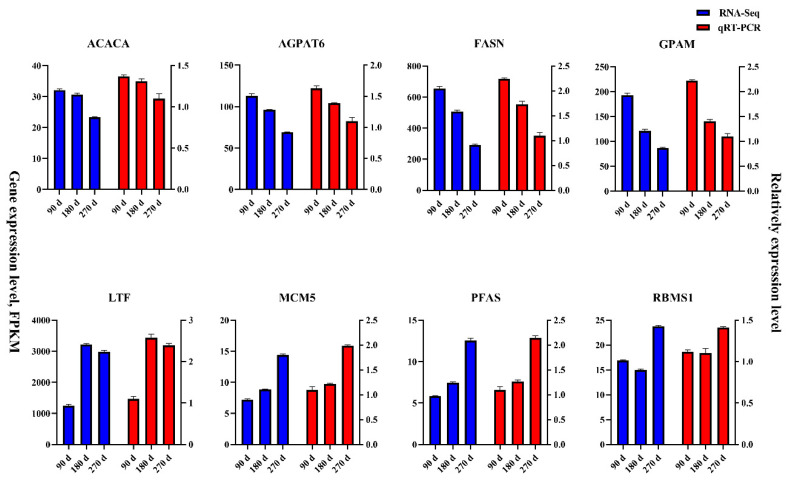
Expression levels of 10 differentially expressed genes detected by qRT-PCR and transcriptome sequencing.

qRT-PCR was conducted to validate the expression levels of acetyl-CoA carboxylase alpha (*ACACA*), glycerol-3-phosphate acyltransferase 4 (*AGPAT6*), fatty acid synthase (*FASN*), glycerol-3-phosphate acyltransferase, mitochondrial (*GPAM*), lactotransferrin (*LTF*), minichromosome maintenance complex component 5 (*MCM5*), phosphoribosylformylglycinamidine synthase (*PFAS*), and RNA binding motif single stranded interacting protein 1 (*RBMS1*). The raw data of the qRT-PCR are shown in File S1. The expression patterns of these eight genes were consistent with the transcriptome data (Figure 7). Moreover, the Pearson correlation coefficient (R) was 0.985 between the sequencing data and qRT-PCR results, which demonstrated that the transcripts in the sequencing data were highly consistent with those in the qRT-PCR results.

## 4. Discussion

### 4.1. Differentially Expressed Genes during Peak and Mid-Lactation

A total of 882 DEGs were identified at mid-lactation compared with peak lactation. There were 11 significant terms related to cell apoptosis and 5 significant terms related to cell proliferation and negative regulation of the apoptotic process at mid-lactation compared to peak lactation (Table S9). Moreover, daily milk yield displayed a significant decrease from peak to mid-lactation. According to the current data, the acinar area at peak lactation was three times that at mid-lactation. The number of cells per acinus at peak lactation was significantly higher than that at mid-lactation. Histological observations showed that the number of mammary gland cells is an important factor determining milk production, which was consistent with a previous study [38]. Obviously, apoptosis was observed in the mammary gland cells of Holstein dairy cows at mid-lactation, which was the reason why the daily milk yield decreased. In addition, Ollier et al. [39], for the first time, analyzed transcriptome to study the impact of nutrition on ruminant mammary gene expression and demonstrated that food deprivation alters mammary transcriptome simultaneously to milk production and composition. Most genes influenced by food deprivation are downregulated, among which genes are involved in the drop in milk production and milk component secretion. Therefore, Ollier et al. [39] believed that certain genes could participate in slowing proliferation and differentiation of mammary cells in response to food deprivation as well as an orientation of mammary cells toward programmed cell death, which could correspond to an early step in mammary gland involution.

A total of 11 terms, including 51 DEGs, played roles in material binding at mid-lactation relative to peak lactation (Table S10). The 11 terms were monocarboxylic acid binding (GO:0033293), retinoid binding (GO:0005501), fatty acid binding (GO:0005504), carboxylic acid binding (GO:0031406), organic acid binding (GO:0043177), NADP binding (GO:0050661), glycosaminoglycan binding (GO:0005539), carbohydrate binding (GO:0030246), protein-containing complex binding (GO:0044877), Ras GTPase binding (GO:0017016), and small GTPase binding (GO:0031267). Because most short- and medium-chain FAs are synthesized de novo [7], it is essential for certain genes to traffic intracellular fatty acids. Indeed, the decline in total milk fat signified decreased efficiency of intracellular fatty acid trafficking in mid-lactation versus peak lactation. A factor for the decrease in total milk fat may be that fatty acid-binding protein 4 (*FABP4*) and fatty acid-binding protein 5 (*FABP5*) are downregulated during mid- versus peak lactation. *FABP4* and *FABP5* were enriched in the monocarboxylic acid-binding term and fatty acid-binding term. In addition, another gene in the fatty acid-binding protein family, fatty acid-binding protein 3 (*FABP3*), was found to play an important role in the mammary gland of the Holstein dairy cow from non-lactation to lactation.

Energy-metabolism-related genes adiponectin (*ADIPOQ*), fructose-bisphosphatase 1 (*FBP1*), and galactose mutarotase (*GALM*) showed higher expression levels at peak lactation than at mid-lactation. Our finding was in line with the point of previous studies: these genes might help supply energy for lactation [7,40].

Unlike previous studies [7,8,12,36], we identified an upregulated gene, lactotransferrin (*LTF*), at mid-lactation compared with peak lactation. Casein and whey protein account for more than 95% of the milk proteins in livestock, and lactoferrin falls into the category of whey protein [41,42]. The protein coded by *LTF* is a multifunctional iron-binding glycoprotein of the transferrin family in most mammalian exocrine secretions [43]. This protein is a major iron-binding protein with antimicrobial activity in milk, making it an important component of the non-specific immune system. Upregulated *LTF* is beneficial to immune protein synthesis in milk secreted at mid-lactation.

### 4.2. Differentially Expressed Genes during Peak and Late Lactation

In the peak vs. late group, a total of 1439 DEGs (*p*-value ≤ 0.05) were identified. A *p*-value ≤ 0.05 is considered statistically significant, both past and present [7,44,45,46,47,48,49]. Recently years some studies identified statistical significances using FDR [50,51,52]. In the coming years, FDR will increasingly be applied to assess the significances of data. The mammary gland undergoes periodic cycles of growth, differentiation, and regression throughout adult life [53]. In the peak vs. late group, significant terms were mainly associated with cell apoptosis and energy metabolism (Tables S11 and S12), and only two significant terms related to cell proliferation (Table S11). Our histological observations suggested that the number of cells per acinus at mid-lactation was significantly higher than that at late lactation. Marti et al. [54] thought that the apoptosis and decrease in the number of mammary gland cells are the regression characteristics of mammary glands in late lactation. In our data, *PRL* expression was observed 14.72% reduction from peak to mid-lactation (*p*-value = 0.16), and a 23.41% decrease in late lactation compared with mid-lactation (*p*-value = 0.01). Lacasse et al. [55] support prolactin (PRL) as galactopoietic in dairy cows. Research from Ollier et al. [56] indicated that PRL-release inhibition is a new alternative, which could reduce milk production before drying off and accelerate mammary gland degeneration without disturbing the metabolism of dairy cows. Based on nutrient levels, a study discovered that dietary supplementation of unsaturated fatty acid (UFA) upregulated the gene sets associated to cell development, apoptosis, remodeling, nutrient metabolic process, as well as immune system response predominantly [57]. Another study showed that supplementing unprotected dietary UFA not only affects the remodeling and immune functions of the bovine mammary gland but also holds potential repercussions for its integrity and health, and milk quality [58]. In the current study, another feature of late lactation was that the energy consumed by dairy cows was different between peak and late lactation.

### 4.3. Differentially Expressed Genes Related in Milk Fat Synthesis

A STEM analysis explored the expression pattern of the downregulated DEGs in the bovine mammary gland during lactation decline. The results illustrated that nine GO terms and eight KEGG pathways, including 17 genes, were associated with milk fat synthesis (Table S8). In other words, the expression levels of the genes related to milk fat synthesis were downregulated as dairy cows entered mid- and late lactation.

Milk fat synthesis occurs in the mammary gland epithelial cells (BMECs). The main component of milk fat is triglycerides, glycerol esters with three fatty acids [59]. Fatty acids used for milk fat synthesis mainly comes from two pathways: exogenous intake and endogenous synthesis [40,60,61]. In the endogenous synthesis pathway, fatty acids need to be transported into BMECs. The CD36 molecule (*CD36*) attracts fatty acids close to the outer plasma membrane and lowers the fatty acid activation energy required for passage through the lipid bilayer, thereby accelerating cellular fatty acid uptake [62]. Endogenous fatty acid biosynthesis is initiated when acetyl-CoA condenses with CO_2_ via acetyl-CoA carboxylase (ACC) to produce malonyl-CoA [63]. Furthermore, acetyl-CoA carboxylase alpha (*ACACA*) is considered a rate-limiting enzyme in de novo fatty acid synthesis in bovine mammary glands [64]. Fatty acid synthase (*FASN*) is another crucial enzyme in endogenous lipogenesis in mammals, and it catalyzes long-chain fatty acid synthesis [65]. The expression patterns of *CD36*, *ACACA,* and *FASN* were consistent with the findings of Bionaz and Loor [40].

Unlike previous studies [8,27,40], our data support a point of view that in the de novo glycerolipid synthesis pathway, the first step is the synthesis of lysophosphatidic acids (LPAs); then, LPAs form phosphatidic acids via the catalytic action of *AGPAT*. Phosphatidic acids are the precursors for triglyceride biosynthesis. This view suggests that *AGPAT1* and *AGPAT6* are candidate genes involved in milk fat synthesis.

### 4.4. Differentially Expressed Genes Related in Milk Protein Synthesis

Daily milk yield and the production of fat protein displayed a declining trend due to the inhibition of cell proliferation and the cell cycle. Moreover, the significant terms were mainly related to protein synthesis in Profile 12 (Table S8), which might account for the rising milk protein concentration from peak to late lactation.

In cells’ life process, a protein molecule with biological activity receives genetic information in deoxyribonucleic acid (DNA) through transcription and translation [66]. Milk protein is no exception. Adenosine monophosphate (AMP) is necessary as monomer units of ribonucleic acid (RNA) in the transcription process [67]. The upregulation of adenylate kinase 2 (*AK2*) provides more AMP for transcription [68,69] to favor milk protein synthesis. Protein synthesis is initiated by transcription into a pre-messenger RNA (pre-mRNA); then, the pre-mRNA undergoes pre-mRNA splicing and processing to generate mature mRNA [70,71]. In the current study, five significant terms, including 15 genes in Profile 12, participated in the mature mRNA process; these were the mRNA processing term (GO:0006397), RNA processing term (GO:0006396), RNA splicing term (GO:0008380), mRNA splicing term (GO:0000398) and regulation of mRNA splicing term (GO:0048024) (Table S8).

Translation contains two processes. One is when mature mRNAs are transported from the nucleus to the cytoplasm. The other is when the ribosome synthesizes proteins according to genetically encoded information in these mature mRNAs [72]. Eukaryotic ribosomes, responsible for the translation of protein, consisting of two subunits: small (40S) and large (60S) subunits. Each subunit is composed of ribosomal RNA (rRNA) and protein [73,74,75]. In Profile 12, a total of five significant terms were involved in the formation of ribosomes in the peak vs. late group (Table S8). Terms contributing to protein synthesis were the five significant terms (including 15 DEGs): ribonucleoprotein complex biogenesis term (GO:0022613), ribonucleoprotein complex assembly term (GO:0022618), ribosome biogenesis term (GO:0042254), ribosomal large subunit biogenesis term (GO:0042273), and rRNA processing term (GO:0006364). The precursors of the two ribosomal subunits must exit the nucleus to function when translation occurs in the cytoplasm [76]. This process in bovine mammary glands from peak to late lactation is accomplished in part by the DEGs (*RRS1*, *SDAD1*, *SHFM1,* and *SRSF3*) in the ribonucleoprotein complex export from nucleus term (GO:0071426), ribosomal large subunit export from nucleus term (GO:0000055) and spliceosomal snRNP assembly term (GO:0000387). A chain of amino acids is commonly referred to as the primary structure of a protein [77]. Protein folding is necessary for its maturation [78]. Seven DEGs (*CCT3*, *CCT4*, *CCT6A*, *HSP90AA1*, *HSP90AB1*, *HSP90B1,* and *HSPE1*) made an important contribution to protein maturation in the mammary glands of Holstein dairy cows from peak to late lactation. The above seven genes were significantly enriched in the protein folding term (GO:0006457), “de novo” protein folding term (GO:0006458), and chaperone-mediated protein folding term (GO:0061077).

## 5. Conclusions

This is the first study that has focused on the variation of gene expression profiles in the dairy cow mammary gland using transcriptome sequencing based on the STEM clustering method in lactation decline. Five significant gene expression profiles were obtained in this process. The results indicate that both the number and the biological activity of mammary gland cells decreased, and the DEGs associated with protein synthesis were upregulated. These findings provide a foundation for future studies on the molecular mechanisms underlying mammary gland development in dairy cows.

## Figures and Tables

**Table 1:** Milk yield, milk composition, and somatic cell count of the three cows at the 90th, 180th, and 270th day after lactation (mean ± SD).

Lactation Days	90 Days	180 Days	270 Days	*p*-Value
Milk yield (kg)	34.52 ± 1.72 ^a^	31.23 ± 1.53 ^b^	26.37 ± 1.21 ^c^	<0.05
Milk protein (g/100 g)	3.05 ± 0.07 ^c^	3.27 ± 0.05 ^b^	3.53 ± 0.06 ^a^	<0.05
Milk protein (g/day)	1053.16 ± 29.25	1023.87 ± 17.39	931.27 ± 20.11	>0.05
Milk fat (g/100 g)	3.26 ± 0.08 ^c^	3.61 ± 0.07 ^b^	3.99 ± 0.08 ^a^	<0.05
Milk fat (g/day)	1124.96 ± 28.63	1126.79 ± 26.18	1053.36 ± 20.46	>0.05
Lactose (g/100 g)	5.24 ± 0.10 ^a^	4.98 ± 0.09 ^b^	4.70 ± 0.07 ^c^	<0.05
Lactose (g/day)	1809.13 ± 42.17	1556.46 ± 22.65	1240.23 ± 28.62	<0.05
Somatic cell count (SCC) (10^4^/mL)	23.82	32.57	47.22	-
Somatic cell score (SCS)	4.25 ± 0.02 ^c^	4.70 ± 0.03 ^b^	5.24 ± 0.02 ^a^	<0.05

Note: ^a,b,c^ = *p* < 0.05 in the row (lactation days effect). The original data for SCC was transformed using SCS = log_2_ (SCC/100,000) + 3 for variance analysis reported as back-transformed.

**Table 2:** The acinus area, lipid droplets area, and number of nuclei in an acinus.

Lactation Days	90 Days	180 Days	270 Days	*p*-Value
Average acinus area (μm^2^)	378,109 ± 43,965 ^a^	154,808 ± 16,671 ^b^	28,361 ± 2107 ^c^	<0.05
The number of nuclei per acinus	42.50 ± 2.12 ^a^	20.50 ± 2.12 ^b^	13.50 ± 0.71 ^c^	<0.05
The average area of lipid droplets (μm^2^)	1348.73 ± 56.33	1336.74 ± 69.80	1355.03 ± 35.65	>0.05

Note: ^a,b,c^ = *p* < 0.05 in the row (lactation days effect).

## Data Availability

The raw sequence data have been deposited in the Genome Sequence Archive in the BIG Data Center, Beijing Institute of Genomics (BIG), Chinese Academy of Sciences, under accession number CRA002742 and are publicly accessible at http://bigd.big.ac.cn/gsa (6 June 2020).

## Supplementary Materials

The following are available online at https://www.mdpi.com/article/10.3390/genes12060942/s1, Table S1: Milk yield, milk composition, and somatic cell count of the 33 cows in the 90th, 180th, and 270th day after lactation. Table S2: The primers used for qRT-PCR. Table S3: Basic information of sequencing reads and bases. Table S4: Top 20 significantly enriched gene ontology (GO) terms in each category during peak and mid-lactation. Table S5: Top 20 significantly enriched gene ontology (GO) terms in each category during peak and late lactation. Table S6: Key significantly enriched gene ontology (GO) terms in profile 15. Table S7: Significantly enriched gene ontology (GO) terms and KEGG pathway related to milk fat synthesis in profile 0. Table S8: Significantly enriched gene ontology (GO) terms related to protein synthesis in profile 12. Table S9: Significantly enriched gene ontology (GO) terms related to cell proliferation and apoptotic during peak and mid-lactation. Table S10: Significantly enriched gene ontology (GO) terms related to material binding during peak and mid-lactation. Table S11: Significantly enriched gene ontology (GO) terms related to cell apoptosis and proliferation during peak and late lactation. Table S12: Significantly enriched gene ontology (GO) terms related to energy metabolism during peak and late lactation. Figure S1: The acinar area statistics. Figure S2: Principal component analysis of the gene expression profiles. Figure S3: Gene ontology (GO) functional enrichment analysis of differentially expressed genes (DEGs). Figure S4: Kyoto Encyclopedia of Genes and Genomes (KEGG) pathway enrichment analysis of differentially expressed genes (DEGs). File S1: The statistical data of acinar area, lipid droplet area, and cell nucleus numbers.
