# Supplementary material for: Short Time-Series Expression Transcriptome Data Reveal the Gene Expression Patterns of Dairy Cow Mammary Gland as Milk Yield Decreased Process
Source: Genes (Basel). 2021 Jun 20;12(6):942. doi: 10.3390/genes12060942 (PMC8235497; doi:10.3390/genes12060942)
Supplement: Supplementary file 1 [file genes-12-00942-s001.zip › genes-1166096-supplementary/supplementary materials/Supplementary figures.pdf]

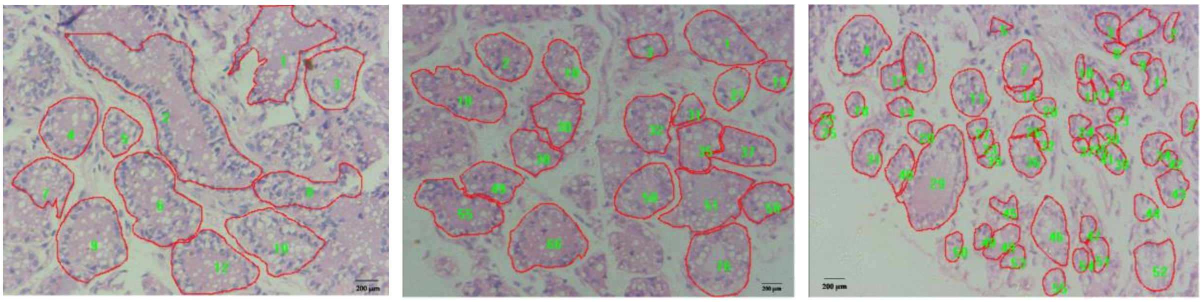

(a) (b) (c)

Figure S1. The acinar area statistics. (a) The acinar area at 90 d of lactation. (b) The acinar area at 180 d of lactation. (c) The acinar area at 270 d of lactation.

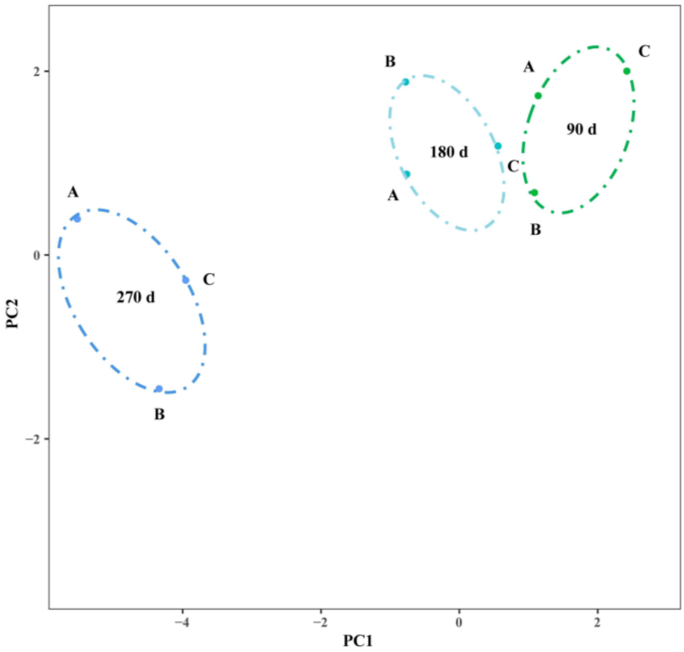

Figure S2. Principal component analysis of the gene expression profiles.

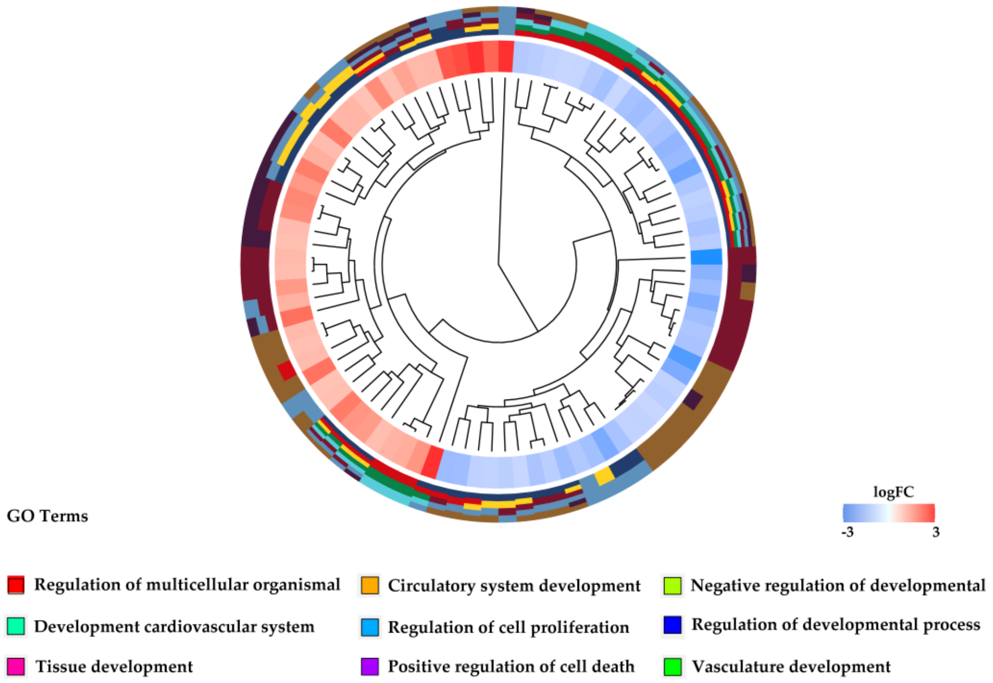

(a)

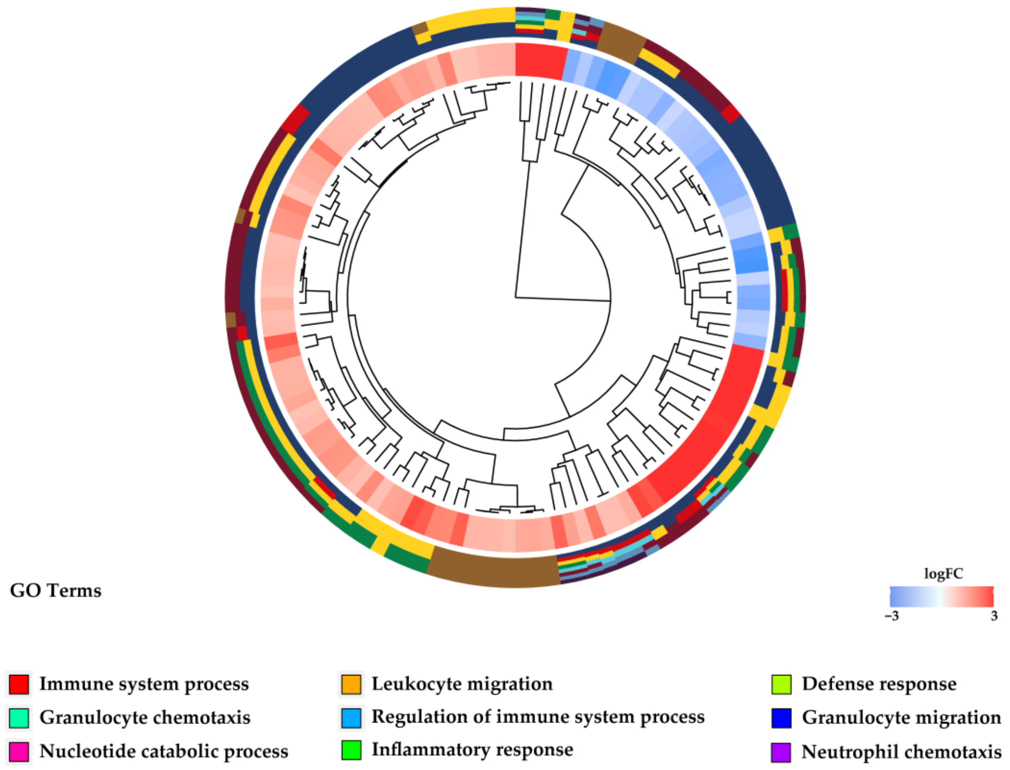

(b)

Figure S3. Gene ontology (GO) functional enrichment analysis of differentially expressed genes (DEGs). Cluster graph summarizing selected features of DEGs enriched in top nine terms in (a) 90 d vs 180 d group and (b) 90 d vs 270 d group.

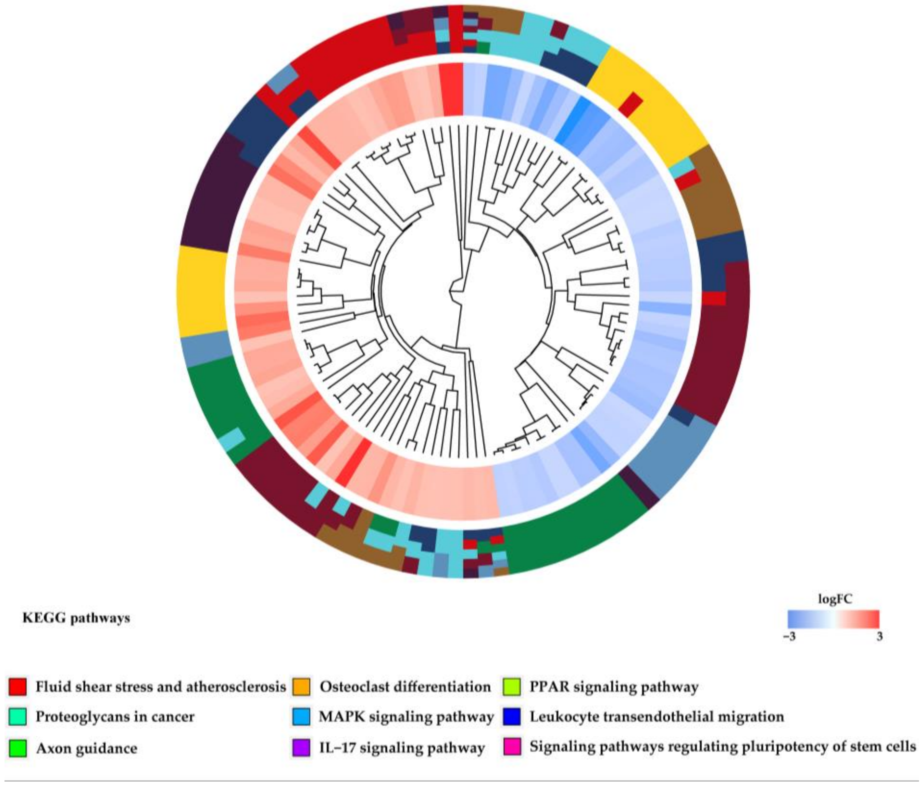

(a)

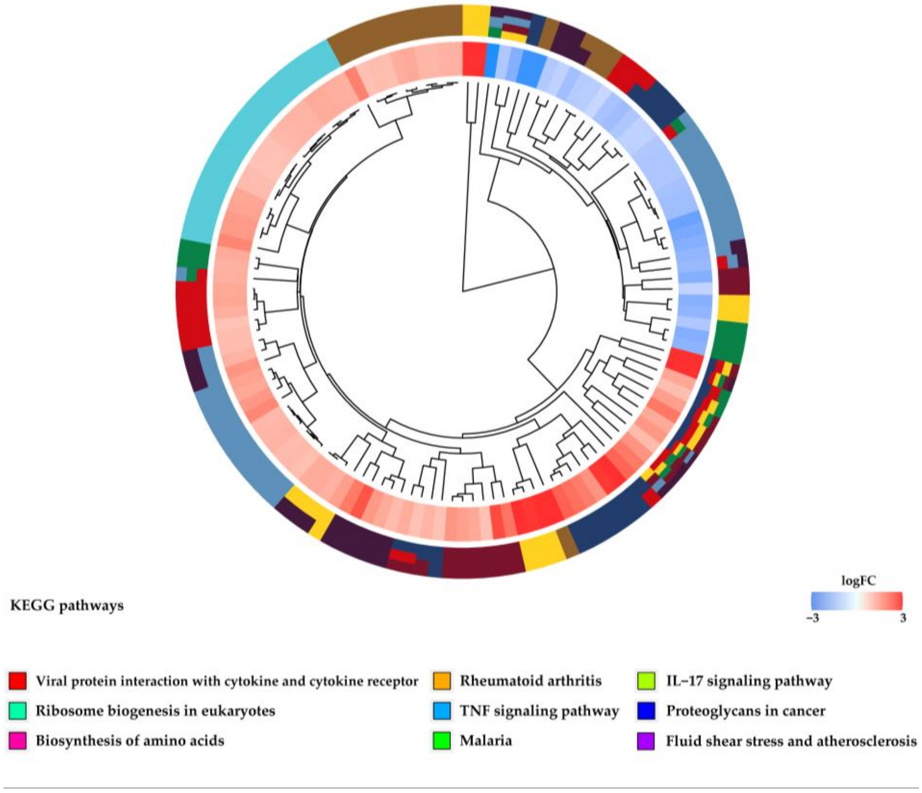

(b)

Figure S4. Kyoto Encyclopedia of Genes and Genomes (KEGG) pathway enrichment analysis of differentially expressed genes (DEGs). Cluster graph summarizing selected features of DEGs enriched in top nine KEGG pathways in (a) 90 d vs 180 d group and (b) 90 d vs 270 d group.
