# Supplementary material for: Short Time-Series Expression Transcriptome Data Reveal the Gene Expression Patterns of Dairy Cow Mammary Gland as Milk Yield Decreased Process
Source: Genes (Basel). 2021 Jun 20;12(6):942. doi: 10.3390/genes12060942 (PMC8235497; doi:10.3390/genes12060942)
Supplement: Supplementary file 1 [file genes-12-00942-s001.zip › genes-1166096-supplementary/supplementary materials/Table S1. The primers used to qRT-PCR..pdf]

Table S1. The primers used to qRT-PCR.

Gene	Forward Primers (5′-3′)	Reverse Primers (5′-3′)	Length (bp)	GenBank ID
ACACA	CTCCAACCTCAACCACTACGG	GGGGAATCACAGAAGCAGCC	171	XM_005693156.1
AGPAT6	AAGCAAGTTGCCCATCCTCA	AAACTGTGGCTCCAATTTCGA	101	NM_001083669.1
FASN	GGGCTCCACCACCGTGTTCCA	GCTCTGCTGGGCCTGCAGCTG	226	DQ915966.3
GPAM	ATTGACCCTTGGCACGATAG	AACAGCACCTTCCCACAAAG	188	AY515690
LTF	CGTGAGGACTTCAGGTTGCT	CTATCGCTCCGAGACACCAC	113	NM_180998.2
MCM5	TCATCCAGGACTTCACCAAGC	TGCATGCGGTGTTGGATCTC	97	NM_001075290.2
PFAS	CCTACAGGAGTAGCCCCCTT	TTGGATCCTCCCAGGGCATA	163	NM_001256564.1
RBMS1	GGAGAGGCTGGAATGACACT	CTTTGCACCTGGTAGGCAGA	158	NM_001035361.1
RPS9	CCTCGACCAAGAGCTGAAG	CCTCCAGACCTCACGTTTGTTC	62	NM_001034034.2
β-actin	CATCCTGACCCTCAAGTA	CTCGTTGTAGAAGGTGTG	91	NM_173979.3
