# Supplementary material for: Short Time-Series Expression Transcriptome Data Reveal the Gene Expression Patterns of Dairy Cow Mammary Gland as Milk Yield Decreased Process
Source: Genes (Basel). 2021 Jun 20;12(6):942. doi: 10.3390/genes12060942 (PMC8235497; doi:10.3390/genes12060942)
Supplement: Supplementary file 1 [file genes-12-00942-s001.zip › genes-1166096-supplementary/supplementary materials/Table S10. Significantly enriched gene ontology (GO) terms related to material binding during peak and mid lactation.pdf]

Table S10. Significantly enriched gene ontology (GO) terms related to material binding during peak and mid lactation.

Category	ID	Term	*P*-value	Gene Name	Number of Genes
molecular function	GO:0033293	monocarboxylic acid binding	< 0.001	*ALOX5AP, PAEP, SERPINA5, CRABP1, FABP4, FABP5, S100A9*	7
molecular function	GO:0005501	retinoid binding	0.001	*PAEP, SERPINA5, CRABP1, PTGDS, FABP5*	5
molecular function	GO:0005504	fatty acid binding	0.002	*ALOX5AP, PAEP, FABP4, FABP5, S100A9*	5
molecular function	GO:0031406	carboxylic acid binding	0.002	*ALOX5AP, PAEP, SERPINA5, CRABP1, FABP4, FABP5, ADIPOQ, TAT, S100A9*	9
molecular function	GO:0043177	organic acid binding	0.002	*ALOX5AP, PAEP, SERPINA5, CRABP1, FABP4, FABP5, ADIPOQ, TAT, S100A9*	9
molecular function	GO:0050661	NADP binding	0.003	*NOS3, DPYD, CRYM, POR, FMO3*	5
molecular function	GO:0005539	glycosaminoglycan binding	0.013	*PGLYRP1, THBS4, CXCL10, SERPINA5, APOE, CLEC3B, LTF, LTBP2*	8
molecular function	GO:0030246	carbohydrate binding	0.014	*GALNT6, CHI3L1, CLEC1A, SLC2A5, COLEC12, SBSPON, CLEC3B, GALM, FBP1*	9
molecular function	GO:0044877	protein-containing complex binding	0.015	*ADRB2, FCGR2B, MAPT, SLC6A4, CORO1A, CFL2, THBS4, TGFBI, USH1C, CTSK, FAP, CORO2A, PIM1, SCIN, COLEC12, FERMT3, TP53, MYC, ZC3H12A*	19
molecular function	GO:0017016	Ras GTPase binding	0.045	*SLC6A4, SGSM3, VAV1, ARHGEF39, CAV1, RASGEF1B*	6
molecular function	GO:0031267	small GTPase binding	0.045	*SLC6A4, SGSM3, VAV1, ARHGEF39, CAV1, RASGEF1B*	6
