# Supplementary material for: Short Time-Series Expression Transcriptome Data Reveal the Gene Expression Patterns of Dairy Cow Mammary Gland as Milk Yield Decreased Process
Source: Genes (Basel). 2021 Jun 20;12(6):942. doi: 10.3390/genes12060942 (PMC8235497; doi:10.3390/genes12060942)
Supplement: Supplementary file 1 [file genes-12-00942-s001.zip › genes-1166096-supplementary/supplementary materials/Table S11. Significantly enriched gene ontology (GO) terms related to cell apoptosis and proliferation during peak and late lactation.pdf]

Table S11. Significantly enriched gene ontology (GO) terms related to cell apoptosis and proliferation during peak and late lactation.

Category	ID	Term	*P*-value	Gene Name	Number of Genes
biological process	GO:0010942	positive regulation of cell death	< 0.001	*S100A9, IL6, MMP9, TGFB2, C1QBP, PPID, PYCARD, CD40, RNF183, TNF, SCIN, S100A8, PDIA3, ZC3H12A, CCL3, RAPGEF2, TP53, CCL5, F2R, SHQ1, ITM2C, CCL2, C1QA, PPIF, MAPT, FAP, PLAUR, MYC, CLU, PPARG*	30
biological process	GO:0043065	positive regulation of apoptotic process	< 0.001	*S100A9, IL6, MMP9, TGFB2, C1QBP, PPID, PYCARD, CD40, RNF183, TNF, SCIN, S100A8, PDIA3, ZC3H12A, CCL3, RAPGEF2, TP53, CCL5, F2R, SHQ1, ITM2C, CCL2, PPIF, FAP, PLAUR, MYC, CLU, PPARG*	28
biological process	GO:0043068	positive regulation of programmed cell death	< 0.001	*S100A9, IL6, MMP9, TGFB2, C1QBP, PPID, PYCARD, CD40, RNF183, TNF, SCIN, S100A8, PDIA3, ZC3H12A, CCL3, RAPGEF2, TP53, CCL5, F2R, SHQ1, ITM2C, CCL2, PPIF, FAP, PLAUR, MYC, CLU, PPARG*	28
biological process	GO:0010941	regulation of cell death	0.001	*S100A9, NOC2L, IL6, MMP9, HSP90AB1, TNFAIP8L2, BCL2A1, DNMT1, TGFB2, C1QBP, PPID, ANGPTL4, PYCARD, CD40, SOCS3, RSL1D1, RNF183, TNF, SCIN, S100A8, PDIA3, ZC3H12A, ANGPT1, CCL3, RAPGEF2, HSP90B1, GPR37L1, TXN, APEX1, TP53, CCL5, F2R, SHQ1, ITM2C, FCER1G, PSMG2, CCL2, C1QA, PPIF, CITED2, MAPT, FAP, PLAUR, MYC, SLC25A5, TNFAIP8L1, LTF, CL2L15, CLU, PPARG*	50
biological process	GO:0008219	cell death	0.001	*S100A9, NOC2L, IL6, MMP9, CASP4, HSP90AB1, TNFAIP8L2, BCL2A1, GABARAP, DNMT1, IFI27L2, TGFB2, C1QBP, CDCA7, PPID, ANGPTL4, ETV6, CHI3L1, THEM4, PYCARD, CD40, SOCS3, XAF1, RSL1D1, RNF183, TNF, SCIN, S100A8, PDIA3, PDCL3, ZC3H12A, ANGPT1, CCL3, RAPGEF2, HSP90B1, GPR37L1, TXN, APEX1, HMOX1, CDIP1, TP53, CCL5, S100A14, F2R, SHQ1, ITM2C, FCER1G, PMP22, PSMG2, CCL2, C1QA, PPIF, CITED2, MAPT, FAP, EYA2, PLAUR, MYC, SLC25A5, TNFAIP8L1, CIDEC, IRF1, LTF, BCL2L15, CLU, PPARG*	66
biological process	GO:0042981	regulation of apoptotic process	0.002	*S100A9, NOC2L, IL6, MMP9, HSP90AB1, TNFAIP8L2, BCL2A1, DNMT1, TGFB2, C1QBP, PPID, ANGPTL4, PYCARD, CD40, SOCS3, RSL1D1, RNF183, TNF, SCIN, S100A8, PDIA3, ZC3H12A, ANGPT1, CCL3, RAPGEF2, HSP90B1, APEX1, TP53, CCL5, F2R, SHQ1, ITM2C, FCER1G, PSMG2, CCL2, PPIF, CITED2, FAP, PLAUR, MYC, SLC25A5, TNFAIP8L1, LTF, BCL2L15, CLU, PPARG*	46
biological process	GO:0006915	apoptotic process	0.002	*S100A9, NOC2L, IL6, MMP9, CASP4, HSP90AB1, TNFAIP8L2, BCL2A1, GABARAP, DNMT1, IFI27L2, TGFB2, C1QBP, CDCA7, PPID, ANGPTL4, ETV6, CHI3L1, THEM4, PYCARD, CD40, SOCS3, XAF1, RSL1D1, RNF183, TNF, SCIN, S100A8, PDIA3, PDCL3, ZC3H12A, ANGPT1, CCL3, RAPGEF2, HSP90B1, APEX1, HMOX1, CDIP1, TP53, CCL5, S100A14, F2R, SHQ1, ITM2C, FCER1G, PSMG2, CCL2, PPIF, CITED2, FAP, EYA2, PLAUR, MYC, SLC25A5, TNFAIP8L1, CIDEC, IRF1, LTF, BCL2L15, CLU, PPARG*	61
biological process	GO:0012501	programmed cell death	0.004	*S100A9, NOC2L, IL6, MMP9, CASP4, HSP90AB1, TNFAIP8L2, BCL2A1, GABARAP, DNMT1, IFI27L2, TGFB2, C1QBP, CDCA7, PPID, ANGPTL4, ETV6, CHI3L1, THEM4, PYCARD, CD40, SOCS3, XAF1, RSL1D1, RNF183, TNF, SCIN, S100A8, PDIA3, PDCL3, ZC3H12A, ANGPT1, CCL3, RAPGEF2, HSP90B1, APEX1, HMOX1, CDIP1, TP53, CCL5, S100A14, F2R, SHQ1, ITM2C, FCER1G, PSMG2, CCL2, PPIF, CITED2, FAP, EYA2, PLAUR, MYC, SLC25A5, TNFAIP8L1, CIDEC, IRF1, LTF, BCL2L15, CLU, PPARG*	61
biological process	GO:0008285	negative regulation of cell proliferation	0.007	*TGFB2, TNF, SCIN, WDR77, RAPGEF2, CD9, TP53, F2R, SERPINF1, DDAH1, CCL2, KRT5, FAP, ENPP3, CAMK2N1, IRF1, CNN1, PTN, PPARG, APOD*	20
biological process	GO:0043280	positive regulation of cysteine-type endopeptidase activity involved in apoptotic process	0.009	*S100A9, PYCARD, TNF, S100A8, F2R, MYC, PPARG*	7
biological process	GO:1904035	regulation of epithelial cell apoptotic process	0.009	*ANGPTL4, CD40, TNF, ANGPT1, CCL2*	5

biological process	GO:1904019	epithelial cell apoptotic process	0.013	*ANGPTL4, CD40, TNF, ANGPT1, CCL2*	5
biological process	GO:0050680	negative regulation of epithelial cell proliferation	0.018	*TGFB2, TNF, WDR77, SERPINF1, CCL2, KRT5, PPARG*	7
biological process	GO:0008283	cell proliferation	0.003	*IL6, MMP9, SERPINF2, IMPDH2, DNMT1, TSPAN1, TGFB2, CDCA7, NDRG1, ETV6, PYCARD, IL18, CD40, THBS4, TNF, SCIN, IL34, ANGPT1, WDR12, WDR77, RAPGEF2, CD9, GPR37L1, GNG5, MARCKSL1, CKS2, TP53, CCL5, JUNB, F2R, IGFBP2, SERPINF1, DDAH1, CNN2, TGFBI, CCL2, PRDX1, KRT5, FAP, FGL1, MYC, F2RL1, ENPP3, SLC25A5, CAMK2N1, PPP1R16B, IRF1, LTF, S1PR1, CNN1, CLU, PTN, PPARG, APOD*	54
biological process	GO:0042127	regulation of cell proliferation	0.004	*IL6, MMP9, SERPINF2, DNMT1, TGFB2, CDCA7, NDRG1, PYCARD, IL18, CD40, THBS4, TNF, SCIN, IL34, ANGPT1, WDR77, RAPGEF2, CD9, GPR37L1, GNG5, MARCKSL1, TP53, CCL5, F2R, IGFBP2, SERPINF1, DDAH1, CNN2, CCL2, KRT5, FAP, MYC, ENPP3, SLC25A5, CAMK2N1, PPP1R16B, IRF1, LTF, S1PR1, CNN1, CLU, PTN, PPARG, APOD*	44
biological process	GO:0008284	positive regulation of cell proliferation	0.037	*IL6, MMP9, SERPINF2, DNMT1, TGFB2, PYCARD, IL18, CD40, THBS4, TNF, IL34, WDR77, GPR37L1, GNG5, MARCKSL1, CCL5, F2R, IGFBP2, MYC, SLC25A5, PPP1R16B, LTF, S1PR1, PTN*	24
biological process	GO:0050678	regulation of epithelial cell proliferation	0.049	*TGFB2, THBS4, TNF, WDR77, CCL5, SERPINF1, CCL2, KRT5, MYC, PPP1R16B, PTN, PPARG*	12
