# Supplementary material for: Short Time-Series Expression Transcriptome Data Reveal the Gene Expression Patterns of Dairy Cow Mammary Gland as Milk Yield Decreased Process
Source: Genes (Basel). 2021 Jun 20;12(6):942. doi: 10.3390/genes12060942 (PMC8235497; doi:10.3390/genes12060942)
Supplement: Supplementary file 1 [file genes-12-00942-s001.zip › genes-1166096-supplementary/supplementary materials/Table S12. Significantly enriched gene ontology (GO) terms related to energy metabolism during peak and late lactation.pdf]

Table S12. Significantly enriched gene ontology (GO) terms related to energy metabolism during peak and late lactation.

Category	ID	Term	*P*-value	Gene Name	Number of Genes
biological process	GO:0019363	pyridine nucleotide biosynthetic process	< 0.001	*ACTN3, PGAM1, EIF6, PGK1, TPI1, ENO1, QPRT, TP53, NAPRT, FBP1, GPD1*	11
biological process	GO:0019362	pyridine nucleotide metabolic process	< 0.001	*ACTN3, PGAM1, EIF6, PGK1, TPI1, ENO1, QPRT, TP53, NAPRT, FBP1, GPD1, MPC1*	12
biological process	GO:0006090	pyruvate metabolic process	< 0.001	*ACTN3, SDS, PGAM1, EIF6, PGK1, TPI1, ENO1, TP53, FBP1, GPD1, MPC1*	11
biological process	GO:0042866	pyruvate biosynthetic process	< 0.001	*ACTN3, SDS, PGAM1, EIF6, PGK1, TPI1, ENO1, TP53, FBP1, GPD1*	10
biological process	GO:0072524	pyridine-containing compound metabolic process	< 0.001	*ACTN3, PGAM1, EIF6, PGK1, TPI1, ENO1, QPRT, TP53, NAPRT, FBP1, GPD1, MPC1*	12
biological process	GO:0019359	nicotinamide nucleotide biosynthetic process	< 0.001	*ACTN3, PGAM1, EIF6, PGK1, TPI1, ENO1, QPRT, TP53, FBP1, GPD1*	10
biological process	GO:0046496	nicotinamide nucleotide metabolic process	< 0.001	*ACTN3, PGAM1, EIF6, PGK1, TPI1, ENO1, QPRT, TP53, FBP1, GPD1, MPC1*	11
biological process	GO:0046031	ADP metabolic process	< 0.001	*ACTN3, PGAM1, EIF6, PGK1, TPI1, ENO1, TP53, AK2, FBP1, GPD1*	10
biological process	GO:0006096	glycolytic process	< 0.001	*ACTN3, PGAM1, EIF6, PGK1, TPI1, ENO1, TP53, FBP1, GPD1*	9
biological process	GO:0006757	ATP generation from ADP	< 0.001	*ACTN3, PGAM1, EIF6, PGK1, TPI1, ENO1, TP53, FBP1, GPD1*	9
biological process	GO:0006732	coenzyme metabolic process	< 0.001	*VNN1, ACTN3, MTHFD1L, PGAM1, EIF6, PGK1, SHMT2, MOCOS, TPI1, ENO1, QPRT, MOCS1, TP53, NAPRT, PCBD1, MTHFR, ACACA, FBP1, GPD1, MPC1*	20
biological process	GO:0006110	regulation of glycolytic process	< 0.001	*ACTN3, PGAM1, EIF6, TP53, FBP1, GPD1*	6
biological process	GO:0009135	purine nucleoside diphosphate metabolic process	< 0.001	*ACTN3, PGAM1, EIF6, PGK1, TPI1, ENO1, TP53, AK2, FBP1, GPD1*	10
biological process	GO:0009179	purine ribonucleoside diphosphate metabolic process	< 0.001	*ACTN3, PGAM1, EIF6, PGK1, TPI1, ENO1, TP53, AK2, FBP1, GPD1*	10
biological process	GO:0009185	ribonucleoside diphosphate metabolic process	< 0.001	*ACTN3, PGAM1, EIF6, PGK1, TPI1, ENO1, TP53, AK2, FBP1, GPD1*	10
biological process	GO:0006733	oxidoreduction coenzyme metabolic process	0.001	*ACTN3, PGAM1, EIF6, PGK1, TPI1, ENO1, QPRT, TP53, NAPRT, FBP1, GPD1, MPC1*	12
biological process	GO:0051196	regulation of coenzyme metabolic process	0.001	*ACTN3, PGAM1, EIF6, TP53, FBP1, GPD1*	6
biological process	GO:0043436	oxoacid metabolic process	0.001	*UGDH, DDC, VNN1, ACTN3, ACSBG1, SDS, MTHFD1L, PGAM1, THEM4, EIF6, PGK1, SLC1A3, IVD, SHMT2, ENOSF1, LTA4H, ANGPT1, TPI1, IDH3A, ENO1, PTGDS, GAMT, TP53, HAGH, DPYD, HADHB, DDAH1, AHCY, GOT2, ASRGL1, GLUL, DCN, MDH2, ITIH5, LDHA, FAH, MTHFR, ACACA, FADS2, FBP1, ASS1, GCAT, FASN, SCD5, GPD1, PAH, MPC1*	47
biological process	GO:0006801	superoxide metabolic process	0.001	*SOD2, TNF, NCF1, MAPT, NRROS, F2RL1*	6
biological process	GO:2001169	regulation of ATP biosynthetic process	0.001	*ACTN3, PGAM1, EIF6, TP53, FBP1, GPD1*	6
biological process	GO:0009127	purine nucleoside monophosphate biosynthetic process	0.001	*HPRT1, IMPDH2, ACTN3, PGAM1, EIF6, PGK1, APRT, TPI1, ENO1, TP53, AK2, FBP1, GPD1*	13
biological process	GO:0009168	purine ribonucleoside monophosphate biosynthetic process	0.001	*HPRT1, IMPDH2, ACTN3, PGAM1, EIF6, PGK1, APRT, TPI1, ENO1, TP53, AK2, FBP1, GPD1*	13
biological process	GO:1903578	regulation of ATP metabolic process	0.002	*ACTN3, PGAM1, EIF6, SHMT2, TP53, PPIF, FBP1, GPD1*	8

biological process	GO:0009144	purine nucleoside triphosphate metabolic process	0.041	*ACTN3, PGAM1, EIF6, PGK1, SHMT2, TPI1, ENO1, MOCS1, TP53, AK2, PPIF, SAMHD1, ENPP3, FBP1, GPD1*	15
biological process	GO:0009141	nucleoside triphosphate metabolic process	0.043	*ACTN3, PGAM1, EIF6, DCTPP1, PGK1, SHMT2, TPI1, ENO1, MOCS1, TP53, AK2, PPIF, SAMHD1, ENPP3, FBP1, GPD1*	16
biological process	GO:0009142	nucleoside triphosphate biosynthetic process	0.044	*ACTN3, PGAM1, EIF6, PGK1, TPI1, ENO1, TP53, FBP1, GPD1*	9
biological process	GO:0009161	ribonucleoside monophosphate metabolic process	0.046	*HPRT1, IMPDH2, ACTN3, PGAM1, EIF6, PGK1, SHMT2, APRT, TPI1, ENO1, TP53, AK2, PPIF, ENPP3, FBP1, GPD1*	16
biological process	GO:0009116	nucleoside metabolic process	0.048	*HPRT1, IMPDH2, APRT, MOCS1, DPYD, AHCY, PNP, PTGDR*	8
molecular function	GO:0051287	NAD binding	0.015	*UGDH, ALDH2, IDH3A, AHCY, ALDH1A1, GPD1*	6
molecular function	GO:0016835	carbon-oxygen lyase activity	0.015	*ENOSF1, TPI1, ENO1, APEX1, CA6, PCBD1, FASN, CA4*	8
molecular function	GO:0016836	hydro-lyase activity	0.026	*ENOSF1, ENO1, CA6, PCBD1, FASN, CA4*	6
molecular function	GO:0016829	lyase activity	0.029	*DDC, SDS, MOCOS, ENOSF1, TPI1, ENO1, APEX1, MOCS1, CA6, PCBD1, ECHDC1, FASN, CA4, NPR2*	14
molecular function	GO:0050662	coenzyme binding	0.032	*UGDH, DDC, SDS, CRYM, DDO, IVD, SHMT2, MOCOS, ALDH2, IDH3A, POR, DPYD, AHCY, GOT2, FMO3, ALDH1A1, GCAT, GPD1*	18
molecular function	GO:0050661	NADP binding	0.039	*CRYM, POR, DPYD, FMO3*	4
