# Supplementary material for: Short Time-Series Expression Transcriptome Data Reveal the Gene Expression Patterns of Dairy Cow Mammary Gland as Milk Yield Decreased Process
Source: Genes (Basel). 2021 Jun 20;12(6):942. doi: 10.3390/genes12060942 (PMC8235497; doi:10.3390/genes12060942)
Supplement: Supplementary file 1 [file genes-12-00942-s001.zip › genes-1166096-supplementary/supplementary materials/Table S2. Milk yield and , milk composition and somatic cell count of the 33 cows in the 90th, 180th and 270th day after lactation.pdf]

Table S2. Milk yield, milk composition and somatic cell count of the 33 cows in the 90th, 180th and 270th day after lactation (Mean ± SD).

**Lactation days**	**90 d**	**180 d**	**270 d**	***p-value***
Milk yield (kg)	34.40±1.61 ^{a}	31.12±1.49 ^{b}	26.46±1.15 ^{c}	<0.05
Milk protein (g/100g)	3.03±0.09 ^{c}	3.26±0.08 ^{b}	3.56±0.10 ^{a}	<0.05
Milk protein (g/day)	1043.18±28.37	1015.26±25.91	943.47±24.32	>0.05
Milk fat (g/100g)	3.22±0.06 ^{c}	3.58±0.08 ^{b}	3.97±0.07 ^{a}	<0.05
Milk fat (g/day)	1106.98±21.15	1115.32±23.71	1049.86±19.25	>0.05
Lactose (g/100g)	5.26±0.10 ^{a}	4.96±0.10 ^{b}	4.66±0.08 ^{c}	<0.05
Lactose (g/day)	1808.62±29.64	1544.27±27.25	1235.16±20.73	<0.05
Somatic cell count (SCC) (10^4/mL)	24.03	31.79	46.98	-
Somatic cell score (SCS)	4.26±0.01^{c}	4.67±0.02 ^{b}	5.23±0.01 ^{a}	<0.05

Note: ^{a},^{b},^{c} = $p < 0.05$ in the row (lactation days effect). The original data for SCC was transformed using SCS = log2 (SCC / 100,000) + 3 for variance analysis reported as back-transformed
