# Supplementary material for: Short Time-Series Expression Transcriptome Data Reveal the Gene Expression Patterns of Dairy Cow Mammary Gland as Milk Yield Decreased Process
Source: Genes (Basel). 2021 Jun 20;12(6):942. doi: 10.3390/genes12060942 (PMC8235497; doi:10.3390/genes12060942)
Supplement: Supplementary file 1 [file genes-12-00942-s001.zip › genes-1166096-supplementary/supplementary materials/Table S3. Basic information of sequencing reads and bases.pdf]

Table S3. Basic information of sequencing reads and bases.

Sample	Raw reads	Raw bases	Clean reads	Clean bases	Valid bases	Q30	GC
A-90	61255240	7.66G	60490684	7.56G	98.72%	97.14%	48.50%
B-90	61664866	7.71G	60994408	7.62G	98.89%	97.33%	47.50%
C-90	59050772	7.38G	58314034	7.29G	98.72%	97.11%	49.00%
A-180	73996266	9.25G	72947722	9.12G	98.56%	96.54%	48.00%
B-180	67909316	8.49G	66910040	8.36G	98.51%	96.33%	49.00%
C-180	75935580	9.49G	74705698	9.34G	98.36%	96.19%	50.00%
A-270	71589742	8.95G	70550840	8.82G	98.53%	96.48%	48.50%
B-270	77932606	9.74G	76857188	9.61G	98.60%	96.44%	49.00%
C-270	64104356	8.01G	62867970	7.86G	98.05%	95.92%	49.00%
