# Supplementary material for: Short Time-Series Expression Transcriptome Data Reveal the Gene Expression Patterns of Dairy Cow Mammary Gland as Milk Yield Decreased Process
Source: Genes (Basel). 2021 Jun 20;12(6):942. doi: 10.3390/genes12060942 (PMC8235497; doi:10.3390/genes12060942)
Supplement: Supplementary file 1 [file genes-12-00942-s001.zip › genes-1166096-supplementary/supplementary materials/Table S4. Top 20 significantly enriched gene ontology (GO) terms in each category during peak and mid lactation..pdf]

Table S4. Top 20 significantly enriched gene ontology (GO) terms in each category during peak and mid lactation.

Category	ID	Term	*P*-value	Gene Name	Number of Genes
biological process	GO:2000026	regulation of multicellular organismal development	< 0.001	*HEYL, ADRB2, TMEM106B, MUSTN1, MMP9, TSPAN12, SYT1, MAPT, PI16, KITLG, TEK, TMEM100, CHI3L1, PPARG, DIO3, MEF2C, FKBP4, TNF, GPR4, IL34, MSX2, EGR1, THBS4, IL18, S1PR1, CXCL10, CTSK, ANGPT2, PPP1R16B, NFIB, CAV1, PIM1, C1QA, MT3, SCIN, ITM2C, TP53, GPR37L1, MYC, ZC3H12A, ADIPOQ, CITED2, TMEM98, SDC2, LTF, SEMA3C*	46
biological process	GO:0072359	circulatory system development	< 0.001	*HEYL, TSPAN12, PI16, TEK, TMEM100, CHI3L1, PPARG, GJA4, MEF2C, LOX, GPR4, MSX2, EGR1, THBS4, TGFBI, IL18, S1PR1, CXCL10, FAP, ANGPT2, CLIC4, ANGPTL4, ROM1, PPP1R16B, CAV1, PIM1, TP53, ACTA2, ZC3H12A, CITED2, CDH5, FHL2, B4GALT1, SEMA3C*	34
biological process	GO:0051093	negative regulation of developmental process	< 0.001	*ADRB2, MMP9, MAPT, PI16, SLC6A4, TEK, PPARG, DIO3, FKBP4, TNF, GPR4, MSX2, THBS4, IL18, CXCL10, CTSK, ANGPT2, NFIB, CAV1, MT3, ITM2C, TP53, GPR37L1, MYC, ZC3H12A, ADIPOQ, TMEM98, LTF*	28
biological process	GO:0001944	vasculature development	< 0.001	*TSPAN12, TEK, TMEM100, CHI3L1, PPARG, GJA4, MEF2C, LOX, GPR4, EGR1, THBS4, TGFBI, IL18, S1PR1, CXCL10, FAP, ANGPT2, CLIC4, ANGPTL4, ROM1, PPP1R16B, CAV1, ACTA2, ZC3H12A, CITED2, CDH5, B4GALT1, SEMA3C*	28
biological process	GO:0072358	cardiovascular system development	< 0.001	*TSPAN12, TEK, TMEM100, CHI3L1, PPARG, GJA4, MEF2C, LOX, GPR4, EGR1, THBS4, TGFBI, IL18, S1PR1, CXCL10, FAP, ANGPT2, CLIC4, ANGPTL4, ROM1, PPP1R16B, CAV1, ACTA2, ZC3H12A, CITED2, CDH5, B4GALT1, SEMA3C*	28
biological process	GO:0042127	regulation of cell proliferation	< 0.001	*PYCARD, MUSTN1, MMP9, CAV2, CORO1A, KITLG, CDCA7, PPARG, MEF2C, TNF, IL34, MSX2, EGR1, THBS4, IL18, S1PR1, CXCL10, FAP, DDAH1, RAC2, NDRG1, F2R, PPP1R16B, NFIB, PIM1, CCL5, SCIN, CNN1, CD320, TP53, GPR37L1, FABP4, MYC, CLU, ADIPOQ, CCL2, EPCAM, B4GALT1, LTF*	39
biological process	GO:0050793	regulation of developmental process	< 0.001	*HEYL, ADRB2, TMEM106B, MUSTN1, MMP9, TSPAN12, SYT1, CD44, MAPT, PI16, SLC6A4, CORO1A, KITLG, TEK, BSP5, TMEM100, CHI3L1, PPARG, DIO3, MEF2C, FKBP4, TNF, GPR4, IL34, MSX2, EGR1, THBS4, IL18, S1PR1, CXCL10, CTSK, ANGPT2, RSL1D1, PPP1R16B, NFIB, CAV1, PIM1, C1QA, MT3, SCIN, ITM2C, TP53, GPR37L1, MEDAG, MYC, ZC3H12A, ADIPOQ, CITED2, CCL2, TMEM98, SDC2, LTF, SEMA3C*	53
biological process	GO:0010942	positive regulation of cell death	< 0.001	*PYCARD, MMP9, MAPT, PPARG, MEF2C, TNF, MSX2, FAP, F2R, CAV1, C1QA, MT3, CCL5, SCIN, KCNMA1, ITM2C, TP53, MYC, CLU, ZC3H12A, ADIPOQ, CCL2, S100A9, B4GALT1*	24
biological process	GO:0009888	tissue development	< 0.001	*HEYL, ADRB2, MUSTN1, MMP9, CAV2, ZNF750, SLC44A4, CD44, PI16, KITLG, TMEM100, PPARG, ACTG2, MEF2C, TNF, GPR4, MSX2, EGR1, CFL2, S1PR1, CXCL10, CTSK, ARRDC3, CLIC4, PPP1R16B, NFIB, CAV1, PIM1, SCIN, ANXA2, KCNMA1, TP53, ACTA2, ADIPOQ, CITED2, ASCL2, CLEC3B, SPP1, FHL2, B4GALT1, LTF, SEMA3C, C15H11orf34*	43
biological process	GO:0001568	blood vessel development	< 0.001	*TSPAN12, TEK, TMEM100, CHI3L1, PPARG, GJA4, MEF2C, LOX, GPR4, EGR1, THBS4, TGFBI, IL18, S1PR1, CXCL10, FAP, ANGPT2, ANGPTL4, PPP1R16B, CAV1, ACTA2, ZC3H12A, CITED2, CDH5, B4GALT1, SEMA3C*	26
biological process	GO:0048646	anatomical structure formation involved in morphogenesis	< 0.001	*HEYL, MMP9, TSPAN12, SLC44A4, TEK, TMEM100, CHI3L1, PPARG, MEF2C, TNF, GPR4, MSX2, CFL2, THBS4, TGFBI, IL18, S1PR1, CXCL10, FAP, ANGPT2, ANGPTL4, PPP1R16B, CAV1, PIM1, TP53, ACTA2, ZC3H12A, CITED2, FHL2, B4GALT1, SEMA3C*	31

biological process	GO:0051241	negative regulation of multicellular organismal process	< 0.001	*PYCARD, ADRB2, MMP9, PI16, SLC6A4, TEK, PPARG, DIO3, MEF2C, FKBP4, TNF, GPR4, MSX2, THBS4, CXCL10, CTSK, ARRDC3, ANGPT2, ADORA2B, F2R, NFIB, MT3, KCNMA1, ITM2C, TP53, GPR37L1, MYC, ZC3H12A, PROS1, ADIPOQ, TMEM98, LTF*	32
biological process	GO:0048468	cell development	< 0.001	*HEYL, NEFM, TMEM106B, CAV2, SYT1, SLC44A4, MAPT, PI16, KITLG, TEK, BSP5, PPARG, DIO3, MEF2C, FKBP4, TNF, GPR4, IL34, MSX2, CFL2, CLIC4, NDRG1, PPP1R16B, NFIB, C1QA, MT3, NRROS, KCNMA1, ITM2C, FERMT3, TP53, GPR37L1, ACTA2, ADIPOQ, CITED2, TMEM98, DEFB1, SDC2, FHL2, B4GALT1, LTF, SEMA3C*	42
biological process	GO:0008283	cell proliferation	< 0.001	*PYCARD, MUSTN1, MMP9, CAV2, TSPAN1, CORO1A, KITLG, TEK, CDCA7, PPARG, MEF2C, TNF, IL34, MSX2, EGR1, THBS4, TGFBI, IL18, S1PR1, CXCL10, FAP, CKS2, DDAH1, RAC2, NDRG1, F2R, PPP1R16B, NFIB, PIM1, CCL5, SCIN, CNN1, CD320, TP53, GPR37L1, FABP4, MYC, CLU, ADIPOQ, CCL2, EPCAM, B4GALT1, LTF*	43
biological process	GO:0009653	anatomical structure morphogenesis	< 0.001	*HEYL, TMEM106B, MMP9, TSPAN12, SYT1, SLC44A4, CD44, MAPT, SLC6A4, CORO1A, TEK, TMEM100, CHI3L1, PPARG, ACTG2, DIO3, MEF2C, LOX, TNF, GPR4, MSX2, CFL2, THBS4, TGFBI, IL18, S1PR1, CXCL10, ATP6V1B1, FAP, ANGPT2, CLIC4, ANGPTL4, ROM1, PPP1R16B, NFIB, CAV1, PIM1, MT3, FERMT3, TP53, ACTA2, ZC3H12A, ADIPOQ, CITED2, CCL2, SDC2, FHL2, B4GALT1, LTF, SEMA3C, C15H11orf34*	51
biological process	GO:0008284	positive regulation of cell proliferation	< 0.001	*PYCARD, MUSTN1, MMP9, CAV2, CORO1A, KITLG, MEF2C, TNF, IL34, EGR1, THBS4, IL18, S1PR1, RAC2, F2R, PPP1R16B, PIM1, CCL5, CD320, GPR37L1, FABP4, MYC, EPCAM, B4GALT1, LTF*	25
biological process	GO:0043065	positive regulation of apoptotic process	< 0.001	*PYCARD, MMP9, PPARG, MEF2C, TNF, MSX2, FAP, F2R, CAV1, CCL5, SCIN, KCNMA1, ITM2C, TP53, MYC, CLU, ZC3H12A, ADIPOQ, CCL2, S100A9, B4GALT1*	21
biological process	GO:0043068	positive regulation of programmed cell death	< 0.001	*PYCARD, MMP9, PPARG, MEF2C, TNF, MSX2, FAP, F2R, CAV1, CCL5, SCIN, KCNMA1, ITM2C, TP53, MYC, CLU, ZC3H12A, ADIPOQ, CCL2, S100A9, B4GALT1*	21
biological process	GO:0051240	positive regulation of multicellular organismal process	< 0.001	*PYCARD, HEYL, ADRB2, TMEM106B, MUSTN1, MMP9, SYT1, MAPT, KITLG, TEK, BSP5, TMEM100, CHI3L1, DIO3, MEF2C, TNF, IL34, MSX2, EGR1, IL18, ADORA2B, F2R, PPP1R16B, CAV1, PIM1, C1QA, SCIN, CYBA, GPR37L1, ZC3H12A, FABP5, ADIPOQ, CITED2, AGPAT1, UCP2, TLR3, LTF, SEMA3C*	38
biological process	GO:0072593	reactive oxygen species metabolic process	< 0.001	*NOS3, MAPT, TNF, VAV1, SOD2, DDAH1, TMEM106A, CAV1, MT3, NRROS, CYBA, TP53, NCF1, ZC3H12A*	14
cellular component	GO:0045177	apical part of cell	< 0.001	*ADRB2, CLIC5, SLC1A1, SLC44A4, CD44, SLC6A4, TEK, ATP6V0D2, SCNN1A, ATP6V1B1, FAP, CLIC4, SLC2A5, REEP6, KCNMA1, CD300LG, S100G, SCNN1G, C15H11orf34*	19
cellular component	GO:0016324	apical plasma membrane	< 0.001	*ADRB2, CLIC5, SLC1A1, SLC44A4, CD44, SLC6A4, TEK, ATP6V0D2, SCNN1A, ATP6V1B1, SLC2A5, KCNMA1, CD300LG, S100G, SCNN1G, C15H11orf34*	16
cellular component	GO:0044459	plasma membrane part	< 0.001	*NOS3, ADRB2, CLIC5, CAV2, SLC1A1, TSPAN12, SYT1, SLC44A4, KCNJ2, CD44, MAPT, LIPE, SLC6A4, CORO1A, GJB1, TEK, ATP6V0D2, GJA4, TNF, RHBG, S1PR1, CXCL10, SCNN1A, ATP6V1B1, FAP, SLC8A1, SLC16A1, SLC2A5, ROM1, F2R, CAV1, KCNMA1, CD300LG, CD320, CYBA, GPR37L1, NCF1, S100G, FABP5, SCNN1G, LRRC8C, EPCAM, CLDN4, B4GALT1, RAB26, C15H11orf34*	46
cellular component	GO:0098590	plasma membrane region	< 0.001	*NOS3, ADRB2, CLIC5, CAV2, SLC1A1, SYT1, SLC44A4, CD44, MAPT, LIPE, SLC6A4, TEK, ATP6V0D2, RHBG, SCNN1A, ATP6V1B1, FAP, SLC8A1, SLC2A5, ROM1, F2R, CAV1, KCNMA1, CD300LG, GPR37L1, S100G, FABP5, SCNN1G, CLDN4, B4GALT1, C15H11orf34*	31
cellular component	GO:0005901	caveola	< 0.001	*NOS3, CAV2, LIPE, SLC8A1, F2R, CAV1, KCNMA1*	7

cellular component	GO:0044421	extracellular region part	< 0.001	*PRSS2, MMP9, RNASE6, KITLG, BSP5, CHI3L1, TNF, IL34, THBS4, IL18, ELN, SCNN1A, SERPINA5, FAP, ANGPT2, SERPINA3-1, ANGPTL4, CSN1S2, PTGDS, ANXA2, KCNMA1, APOE, CLU, FABP5, ADIPOQ, CMTM8, TMEM98, DEFB1, CLEC3B, COL12A1, B4GALT1, LTF, LTBP2*	33
cellular component	GO:0005615	extracellular space	< 0.001	*PRSS2, MMP9, RNASE6, KITLG, BSP5, CHI3L1, TNF, IL34, THBS4, IL18, SCNN1A, SERPINA5, FAP, ANGPT2, SERPINA3-1, ANGPTL4, CSN1S2, PTGDS, KCNMA1, APOE, CLU, FABP5, ADIPOQ, CMTM8, TMEM98, CLEC3B, B4GALT1, LTF*	28
cellular component	GO:0044853	plasma membrane raft	< 0.001	*NOS3, CAV2, LIPE, SLC8A1, F2R, CAV1, KCNMA1*	7
cellular component	GO:0098858	actin-based cell projection	0.001	*CD44, KITLG, TEK, ACTG2, USH1C, ATP6V1B1, CLIC4, ACTA2, B4GALT1*	9
cellular component	GO:0045121	membrane raft	0.001	*NOS3, CAV2, MAPT, LIPE, SLC6A4, TEK, TNF, S1PR1, SLC8A1, F2R, CAV1, KCNMA1*	12
cellular component	GO:0098857	membrane microdomain	0.001	*NOS3, CAV2, MAPT, LIPE, SLC6A4, TEK, TNF, S1PR1, SLC8A1, F2R, CAV1, KCNMA1*	12
cellular component	GO:0009986	cell surface	0.002	*TYROBP, TEK, TNF, S1PR1, CXCL10, FAP, CLIC4, TNS1, NRROS, KCNMA1, ADRA2B, ADIPOQ, B4GALT1, LTF*	14
cellular component	GO:0098589	membrane region	0.002	*NOS3, CAV2, MAPT, LIPE, SLC6A4, TEK, TNF, S1PR1, SLC8A1, F2R, CAV1, KCNMA1*	12
cellular component	GO:0005581	collagen trimer	0.003	*C1QA, COLEC12, C1QTNF9, C1QB, ADIPOQ, COL12A1*	6
cellular component	GO:0031226	intrinsic component of plasma membrane	0.004	*SLC1A1, TSPAN12, KCNJ2, SLC6A4, TNF, RHBG, S1PR1, SCNN1A, SLC8A1, SLC16A1, SLC2A5, KCNMA1, CD320, CYBA, NCF1, LRRC8C, CLDN4, RAB26*	18
