# Supplementary material for: Short Time-Series Expression Transcriptome Data Reveal the Gene Expression Patterns of Dairy Cow Mammary Gland as Milk Yield Decreased Process
Source: Genes (Basel). 2021 Jun 20;12(6):942. doi: 10.3390/genes12060942 (PMC8235497; doi:10.3390/genes12060942)
Supplement: Supplementary file 1 [file genes-12-00942-s001.zip › genes-1166096-supplementary/supplementary materials/Table S5. Top 20 significantly enriched gene ontology (GO) terms in each category during peak and late lactation..pdf]

Table S5. Top 20 significantly enriched gene ontology (GO) terms in each category during peak and late lactation.

Category	ID	Term	*P*-value	Gene Name	Number of Genes
cellular component	GO:0034719	SMN-Sm protein complex	< 0.001	*SNRPE, SNRPB, SNRPF, SNRPD2, SMN2, SNRPG, SNRPD1*	7
cellular component	GO:0097525	spliceosomal snRNP complex	< 0.001	*SNRPE, SNRPB, SNRPF, SNRPA, SNRPD2, SNRPG, SNRPC, SNRPD1*	8
cellular component	GO:0034708	methyltransferase complex	< 0.001	*SNRPE, RUVBL2, SNRPB, SNRPF, SNRPD2, WDR77, PRMT5, SNRPG, SNRPD1*	9
cellular component	GO:0030532	small nuclear ribonucleoprotein complex	< 0.001	*SNRPE, SNRPB, SNRPF, SNRPA, SNRPD2, SNRPG, SNRPC, SNRPD1*	8
cellular component	GO:0120114	Sm-like protein family complex	< 0.001	*SNRPE, SNRPB, SNRPF, SNRPA, SNRPD2, SMN2, SNRPG, SNRPC, SNRPD1*	9
cellular component	GO:0046540	U4/U6 x U5 tri-snRNP complex	< 0.001	*SNRPE, SNRPB, SNRPF, SNRPD2, SNRPG, SNRPD1*	6
cellular component	GO:0097526	spliceosomal tri-snRNP complex	< 0.001	*SNRPE, SNRPB, SNRPF, SNRPD2, SNRPG, SNRPD1*	6
cellular component	GO:0005839	proteasome core complex	0.001	*PSMB9, PSMA4, PSMA5, PSMA3, PSMA1, PSMB10, PSMB8*	7
cellular component	GO:0044421	extracellular region part	0.001	*BPIFA1, BPIFB1, PRSS2, IL6, MMP9, SERPINF2, TGFB2, SERPINA3-1, DEFB1, ANGPTL4, CHI3L1, IL18, THBS4, PGK1, LBP, CXCL17, TNF, SCNN1A, IL1B, IL34, BSP5, ANGPT1, CCL3, CD9, MFAP2, PTGDS, CMTM8, ANXA2, FBLN5, MMP2, IGFBP2, RETN, LY96, FAP, DCN, ANGPTL1, CNP, TCN2, CRELD2, FGG, CLIC1, MFAP5, LUM, LTF, MYOC, CRISPLD2, CLU, PTN, APOD, CSN1S2*	50
cellular component	GO:0005615	extracellular space	0.001	*BPIFA1, BPIFB1, PRSS2, IL6, MMP9, SERPINF2, TGFB2, SERPINA3-1, ANGPTL4, CHI3L1, IL18, THBS4, PGK1, LBP, CXCL17, TNF, SCNN1A, IL1B, IL34, BSP5, ANGPT1, CCL3, CD9, PTGDS, CMTM8, FBLN5, IGFBP2, RETN, LY96, FAP, ANGPTL1, CNP, TCN2, CRELD2, FGG, CLIC1, LTF, MYOC, CLU, PTN, APOD, CSN1S2*	42
cellular component	GO:0071007	U2-type catalytic step 2 spliceosome	0.002	*SNRPE, SNRPB, SNRPF, SNRPD2, SNRPG, SNRPD1*	6
cellular component	GO:0031226	intrinsic component of plasma membrane	0.006	*IL6, SLC1A1, RHBG, CD40, SLC1A3, SLC6A6, TNF, SCNN1A, SLC16A1, SLC26A2, HCRTR1, NCF1, FCER1G, SLC8A1, LY96, SLC2A5, C7, SCARA5, RAB26, PLAUR, STRA6, SELL, FOLR3, SLC6A4, S1PR1, CLSTN3, NPR2*	27
cellular component	GO:0034399	nuclear periphery	0.006	*PHB2, NUP93, EIF6, UHRF1, TP53, PCNA, MAPT, KRT8*	8
cellular component	GO:0071005	U2-type precatalytic spliceosome	0.007	*SNRPE, SNRPB, SNRPF, SF3B5, SNRPD2, SNRPG, SNRPD1*	7
cellular component	GO:0071011	precatalytic spliceosome	0.007	*SNRPE, SNRPB, SNRPF, SF3B5, SNRPD2, SNRPG, SNRPD1*	7
cellular component	GO:0042470	melanosome	0.007	*HSP90AB1, HSP90AA1, CCT4, PDIA3, HSP90B1, ANXA2, SERPINF1, AHCY, PDIA4, CTNS, CNP, FASN*	12
cellular component	GO:0048770	pigment granule	0.007	*HSP90AB1, HSP90AA1, CCT4, PDIA3, HSP90B1, ANXA2, SERPINF1, AHCY, PDIA4, CTNS, CNP, FASN*	12
cellular component	GO:0031012	extracellular matrix	0.011	*MMP9, TGFB2, THBS4, MFAP2, ANXA2, FBLN5, MMP2, DCN, MFAP5, LUM, MYOC, CRISPLD2, CLU*	13
cellular component	GO:0009986	cell surface	0.012	*SERPINF2, TYROBP, CD40, SLC1A3, TNF, CD9, CD99L2, GOT2, FAP, SCARA5, NRROS, PLAUR, MXRA8, ENPP3, FOLR3, LTF, S1PR1, ADRA2B*	18
cellular component	GO:0043230	extracellular organelle	0.018	*DEFB1, SCNN1A, IL1B, CD9, ANXA2, CLIC1, MYOC*	7

molecular function	GO:0005102	signaling receptor binding	< 0.001	*CCL20, S100A9, IL6, IL1RN, DOK2, TGFB2, DEFB1, C1QBP, S100A12, PPID, CXCL5, RND1, IL18, THBS4, CXCL2, TNF, S100A8, IL1B, SYT1, TUBB, IL34, FAM49B, ANGPT1, ANGPT4, SPG21, RASL11B, CCL3, RAPGEF2, HSP90B1, CD9, CMTM8, SPADH1, MIA, TP53, CCL5, S100A14, SPP1, PCNA, FBLN5, CXCL8, TGFBI, RETN, LY96, MAPT, FAP, PLAUR, ANGPTL1, FGG, MANF, FERMT3, OGN, LTF, S1PR1, PTN, PPARG*	55
molecular function	GO:0042379	chemokine receptor binding	< 0.001	*CCL20, DEFB1, CXCL5, CCL3, CCL5, S100A14, CXCL8*	7
molecular function	GO:0005126	cytokine receptor binding	0.001	*CCL20, IL6, IL1RN, TGFB2, DEFB1, CXCL5, IL18, TNF, IL34, RASL11B, CCL3, CCL5, S100A14, CXCL8*	14
molecular function	GO:0004298	threonine-type endopeptidase activity	0.001	*PSMB9, PSMA4, PSMA5, PSMA3, PSMA1, PSMB10, PSMB8*	7
molecular function	GO:0070003	threonine-type peptidase activity	0.001	*PSMB9, PSMA4, PSMA5, PSMA3, PSMA1, PSMB10, PSMB8*	7
molecular function	GO:0005178	integrin binding	0.002	*THBS4, IL1B, CD9, FBLN5, TGFBI, FAP, FERMT3*	7
molecular function	GO:0005125	cytokine activity	0.009	*IL6, IL1RN, CXCL5, IL18, TNF, IL1B, IL34, CCL3, CMTM8, SPP1*	10
molecular function	GO:0030971	receptor tyrosine kinase binding	0.009	*DOK2, ANGPT1, ANGPT4, TP53, PCNA*	5
molecular function	GO:0030246	carbohydrate binding	0.010	*LGALS4, CLEC6A, FBXO2, OLR1, CHI3L1, CALR, FCN1, GALM, SLC2A5, COLEC12, ENPP3, SBSPON, SELL, FBP1*	14
molecular function	GO:0050839	cell adhesion molecule binding	0.010	*THBS4, IL1B, CD9, CXADR, FBLN5, TGFBI, FAP, FERMT3*	8
molecular function	GO:0051287	NAD binding	0.015	*UGDH, ALDH2, IDH3A, AHCY, ALDH1A1, GPD1*	6
molecular function	GO:0016835	carbon-oxygen lyase activity	0.015	*ENOSF1, TPI1, ENO1, APEX1, CA6, PCBD1, FASN, CA4*	8
molecular function	GO:1990782	protein tyrosine kinase binding	0.018	*DOK2, ANGPT1, ANGPT4, TP53, PCNA*	5
molecular function	GO:0003714	transcription corepressor activity	0.018	*C1QBP, CRYM, BASP1, PBXIP1, PRMT5, CITED2, BHLHE40*	7
molecular function	GO:0019904	protein domain specific binding	0.020	*HSP90AB1, HSP90AA1, FOXA3, CHAF1A, ETV6, PYCARD, BASP1, SCNN1A, RAPGEF2, GNG5, NCF1, TP53, CXADR, HEYL, CCT6A, SH3BGRL2, MAPT, PLAUR, PPARG, BHLHE40*	20
molecular function	GO:0004930	G protein-coupled receptor activity	0.021	*PROKR2, CXCR1, GPR37L1, HCRTR1, TBXA2R, F2R, GPR68, ADCYAP1R1, ACKR1, AGTR1, PTGDR, F2RL1, S1PR1, ADRA2B*	14
molecular function	GO:0001653	peptide receptor activity	0.022	*PROKR2, CXCR1, GPR37L1, HCRTR1, F2R, AGTR1, F2RL1, NPR2*	8
molecular function	GO:0016836	hydro-lyase activity	0.026	*ENOSF1, ENO1, CA6, PCBD1, FASN, CA4*	6
molecular function	GO:0016829	lyase activity	0.029	*DDC, SDS, MOCOS, ENOSF1, TPI1, ENO1, APEX1, MOCS1, CA6, PCBD1, ECHDC1, FASN, CA4, NPR2*	14
molecular function	GO:0001664	G protein-coupled receptor binding	0.031	*CCL20, DEFB1, C1QBP, CXCL5, CCL3, RAPGEF2, CCL5, S100A14, CXCL8, S1PR1*	10
