# Supplementary material for: Short Time-Series Expression Transcriptome Data Reveal the Gene Expression Patterns of Dairy Cow Mammary Gland as Milk Yield Decreased Process
Source: Genes (Basel). 2021 Jun 20;12(6):942. doi: 10.3390/genes12060942 (PMC8235497; doi:10.3390/genes12060942)
Supplement: Supplementary file 1 [file genes-12-00942-s001.zip › genes-1166096-supplementary/supplementary materials/Table S6. Key significantly enriched gene ontology (GO) terms in profile 15..pdf]

Table S6. Key significantly enriched gene ontology (GO) terms in profile 15.

ID	Term	FDR	Gene Name	Number of Genes
GO:0010941	regulation of cell death	0.004	*CCL2, CCL3, DNMT1, FCER1G, JUNB, LTF, NUP93, PHB2, PPID, RSL1D1, SCIN, TNF, TP53*	13
GO:0051253	negative regulation of RNA metabolic process	0.006	*BHLHE40, DNMT1, IRF1, JUNB, KCTD1, PHB2, PPID, PRMT5, TNF, TP53*	10
GO:2000113	negative regulation of cellular macromolecule biosynthetic process	0.006	*BHLHE40, DNMT1, EIF6, IRF1, JUNB, KCTD1, PHB2, PPID, TNF, TP53*	10
GO:0045892	negative regulation of transcription	0.008	*BHLHE40, DNMT1, IRF1, JUNB, KCTD1, PHB2, PPID, TNF, TP53*	9
GO:0042981	regulation of apoptotic process	0.014	*CCL2, CCL3, DNMT1, FCER1G, LTF, PHB2, PPID, RSL1D1, SCIN, TNF, TP53*	11
GO:0008285	negative regulation of cell population proliferation	0.025	*CCL2, IRF1, PHB2, SCIN, TNF, TP53*	6
GO:1902806	regulation of cell cycle G1/S phase transition	0.026	*CCL2, PHB2, TP53*	3
GO:0031324	negative regulation of cellular metabolic process	0.028	*BHLHE40, DNMT1, EIF6, IRF1, JUNB, KCTD1, LTF, PHB2, PPID, PRMT5, SERPINB1, TNF, TP53*	13
GO:0000291	nuclear-transcribed mRNA catabolic process	0.033	*EXOSC4, POLR2G*	2
GO:0048523	negative regulation of cellular process	0.033	*BHLHE40, CCL2, CCL3, DNMT1, EIF6, FCER1G, GPR171, IRF1, JUNB, KCTD1, LTF, NUP93, PHB2, PPID, PRMT5, SCIN, SERPINB1, TNF, TP53*	19
GO:0000122	negative regulation of transcription by RNA polymerase II	0.034	*BHLHE40, DNMT1, JUNB, PPID, TNF, TP53*	6
GO:0050680	negative regulation of epithelial cell proliferation	0.036	*CCL2, PHB2, TNF*	3
GO:2000134	negative regulation of G1/S transition of mitotic cell cycle	0.040	*CCL2, TP53*	2
GO:0006974	cellular response to DNA damage stimulus	0.036	*KIAA0101, KIF22, MND1, POLD1, RUVBL2, TNF, TP53*	7
