# Supplementary material for: Short Time-Series Expression Transcriptome Data Reveal the Gene Expression Patterns of Dairy Cow Mammary Gland as Milk Yield Decreased Process
Source: Genes (Basel). 2021 Jun 20;12(6):942. doi: 10.3390/genes12060942 (PMC8235497; doi:10.3390/genes12060942)
Supplement: Supplementary file 1 [file genes-12-00942-s001.zip › genes-1166096-supplementary/supplementary materials/Table S7. Significantly enriched gene ontology (GO) terms and KEGG pathway related to milk fat synthesis in profile 0..pdf]

Table S7. Significantly enriched gene ontology (GO) terms and KEGG pathway related to milk fat synthesis in profile 0.

ID	Term / Pathway	FDR	Gene Name	Number of Genes
GO:0008374	O-acyltransferase activity	0.000	*AGPAT1*, *AGPAT6*, *GPAM*, *LRAT*	4
GO:0016747	transferase activity	0.000	*AGPAT1*, *AGPAT6*, *FASN*, *GCAT*, *GPAM*, *LRAT*	6
GO:0016024	CDP-diacylglycerol biosynthetic process	0.006	*AGPAT1*, *AGPAT6*, *GPAM*	3
GO:0046341	CDP-diacylglycerol metabolic process	0.006	*AGPAT1*, *AGPAT6*, *GPAM*	3
GO:0004366	glycerol-3-phosphate O-acyltransferase activity	0.007	*AGPAT6*, *GPAM*	2
GO:0102420	sn-1-glycerol-3-phosphate C16:0-DCA-CoA acyl transferase activity	0.007	*AGPAT6*, *GPAM*	2
GO:0019752	carboxylic acid metabolic process	0.024	*ACACA*, *ASS1*, *C1QTNF9*, *FAH*, *FASN*, *GCAT*, *IVD*, *PAH*	8
GO:0044255	cellular lipid metabolic process	0.027	*ACACA*, *AGPAT1*, *AGPAT6*, *C1QTNF9*, *FASN*, *GPAM*, *LRAT*	7
GO:0008610	lipid biosynthetic process	0.038	*ACACA*, *AGPAT1*, *AGPAT6*, *FASN*, *GPAM*	5
bta00561	Glycerolipid metabolism	0.003	*AGPAT1*, *AGPAT6*, *GPAM*	3
bta00061	Fatty acid biosynthesis	0.004	*ACACA*, *FASN*	2
bta00564	Glycerophospholipid metabolism	0.007	*AGPAT1*, *AGPAT6*, *GPAM*	3
bta01100	Metabolic pathways	0.007	*ACACA*, *AGPAT1*, *AGPAT6*, *ASS1*, *FASN*, *GPAM*, *NT5E*, *UGP2*	7
bta04975	Fat digestion and absorption	0.017	*AGPAT1*, *CD36*	2
bta01212	Fatty acid metabolism	0.018	*ACACA*, *FASN*	2
