# Supplementary material for: Short Time-Series Expression Transcriptome Data Reveal the Gene Expression Patterns of Dairy Cow Mammary Gland as Milk Yield Decreased Process
Source: Genes (Basel). 2021 Jun 20;12(6):942. doi: 10.3390/genes12060942 (PMC8235497; doi:10.3390/genes12060942)
Supplement: Supplementary file 1 [file genes-12-00942-s001.zip › genes-1166096-supplementary/supplementary materials/Table S8. Significantly enriched gene ontology (GO) terms related to protein synthesis in profile 12..pdf]

Table S8. Significantly enriched gene ontology (GO) terms related to protein synthesis in profile 12.

ID	Term	FDR	Gene Name	Number of Genes
GO:0006396	RNA processing	0.000	*C1QBP, MAGOH, MAK16, NOP56, POP5, RPF2, RRS1, SNRPA, SNRPB, SNRPC, SNRPD2, SNRPG, SRSF3, TBL3, TRMT61A*	15
GO:0016071	mRNA metabolic process	0.001	*C1QBP, MAGOH, SNRPA, SNRPB, SNRPC, SNRPD2, SNRPG, SRSF3, TRMT61A, ZC3H12A*	10
GO:0008380	RNA splicing	0.002	*C1QBP, MAGOH, SNRPA, SNRPB, SNRPC, SNRPD2, SNRPG, SRSF3*	8
GO:0006397	mRNA processing	0.004	*C1QBP, MAGOH, SNRPA, SNRPB, SNRPC, SNRPD2, SNRPG, SRSF3*	8
GO:0000398	mRNA splicing	0.013	*SNRPA, SNRPB, SNRPC, SNRPD2, SNRPG*	5
GO:0048024	regulation of mRNA splicing	0.040	*C1QBP, MAGOH, SRSF3*	3
GO:0022613	ribonucleoprotein complex biogenesis	0.000	*C1QBP, HSP90AA1, MAK16, NOC2L, NOP16, NOP56, RPF2, RRS1, SDAD1, SHQ1, SNRPB, SNRPC, SNRPD2, SNRPG, TBL3*	15
GO:0022618	ribonucleoprotein complex assembly	0.000	*C1QBP, HSP90AA1, RPF2, RRS1, SHQ1, SNRPB, SNRPC, SNRPD2, SNRPG*	9
GO:0042254	ribosome biogenesis	0.000	*C1QBP, MAK16, NOC2L, NOP16, NOP56, RPF2, RRS1, SDAD1, TBL3*	9
GO:0042273	ribosomal large subunit biogenesis	0.000	*MAK16, NOC2L, NOP16, RPF2, RRS1, SDAD1*	6
GO:0000387	spliceosomal snRNP assembly	0.003	*SNRPB, SNRPC, SNRPD2, SNRPG*	4
GO:0006364	rRNA processing	0.017	*MAK16, NOP56, RPF2, RRS1, TBL3*	5
GO:0071426	ribonucleoprotein complex export from nucleus	0.006	*RRS1, SDAD1, SHFM1, SRSF3*	4
GO:0000055	ribosomal large subunit export from nucleus	0.015	*RRS1, SDAD1*	2
GO:0006457	protein folding	0.002	*CCT3, CCT4, CCT6A, HSP90AA1, HSP90AB1, HSP90B1, HSPE1*	7
GO:0006458	"de novo" protein folding	0.002	*CCT3, CCT4, CCT6A, HSPE1*	4
GO:0061077	chaperone-mediated protein folding	0.008	*CCT3, CCT4, CCT6A, HSPE1*	4
