# Supplementary material for: Short Time-Series Expression Transcriptome Data Reveal the Gene Expression Patterns of Dairy Cow Mammary Gland as Milk Yield Decreased Process
Source: Genes (Basel). 2021 Jun 20;12(6):942. doi: 10.3390/genes12060942 (PMC8235497; doi:10.3390/genes12060942)
Supplement: Supplementary file 1 [file genes-12-00942-s001.zip › genes-1166096-supplementary/supplementary materials/Table S9. Significantly enriched gene ontology (GO) terms related to cell proliferation and apoptotic during peak and mid lactation.pdf]

Table S9. Significantly enriched gene ontology (GO) terms related to cell proliferation and apoptotic during peak and mid lactation.

Category	ID	Term	*P*-value	Gene Name	Number of Genes
biological process	GO:0042127	regulation of cell proliferation	< 0.001	*PYCARD, MUSTN1, MMP9, CAV2, CORO1A, KITLG, CDCA7, PPARG, MEF2C, TNF, IL34, MSX2, EGR1, THBS4, IL18, S1PR1, CXCL10, FAP, DDAH1, RAC2, NDRG1, F2R, PPP1R16B, NFIB, PIM1, CCL5, SCIN, CNN1, CD320, TP53, GPR37L1, FABP4, MYC, CLU, ADIPOQ, CCL2, EPCAM, B4GALT1, LTF*	39
biological process	GO:0008283	cell proliferation	< 0.001	*PYCARD, MUSTN1, MMP9, CAV2, TSPAN1, CORO1A , KITLG, TEK, CDCA7, PPARG, MEF2C, TNF, IL34, MSX2, EGR1, THBS4, TGFBI, IL18, S1PR1, CXCL10, FAP, CKS2, DDAH1, RAC2, NDRG1, F2R, PPP1R16B, NFIB, PIM1, CCL5, SCIN, CNN1, CD320, TP53, GPR37L1, FABP4, MYC, CLU, ADIPOQ, CCL2, EPCAM, B4GALT1, LTF*	43
biological process	GO:0008284	positive regulation of cell proliferation	< 0.001	*PYCARD, MUSTN1, MMP9, CAV2, CORO1A, KITLG, MEF2C, TNF, IL34, EGR1, THBS4, IL18, S1PR1, RAC2, F2R, PPP1R16B, PIM1, CCL5, CD320, GPR37L1, FABP4, MYC, EPCAM, B4GALT1, LTF*	25
biological process	GO:0050673	epithelial cell proliferation	0.001	*CAV2, TEK, PPARG, MEF2C, TNF, THBS4, FAP, PPP1R16B, NFIB, CCL5, MYC, CCL2, B4GALT1*	13
biological process	GO:0050678	regulation of epithelial cell proliferation	0.004	*CAV2, PPARG, TNF, THBS4, PPP1R16B, NFIB, CCL5, MYC, CCL2, B4GALT1*	10
biological process	GO:0043066	negative regulation of apoptotic process	< 0.001	*NOS3, MMP9, CORO1A, KITLG, TEK, MEF2C, TNF, MSX2, ANGPTL4, F2R, CAV1, PIM1, MT3, CCL5, TP53, MYC, CLU, ZC3H12A, CITED2, CCL2, FHL2, LTF*	22
biological process	GO:0060548	negative regulation of cell death	< 0.001	*NOS3, MMP9, CORO1A, KITLG, TEK, MEF2C, TNF, MSX2, ANGPTL4, F2R, CAV1, PIM1, MT3, CCL5, TP53, GPR37L1, MYC, CLU, ZC3H12A, CITED2, CCL2, FHL2, LTF*	23
biological process	GO:0043069	negative regulation of programmed cell death	< 0.001	*NOS3, MMP9, CORO1A, KITLG, TEK, MEF2C, TNF, MSX2, ANGPTL4, F2R, CAV1, PIM1, MT3, CCL5, TP53, MYC, CLU, ZC3H12A, CITED2, CCL2, FHL2, LTF*	22
biological process	GO:0010942	positive regulation of cell death	< 0.001	*PYCARD, MMP9, MAPT, PPARG, MEF2C, TNF, MSX2, FAP, F2R, CAV1, C1QA, MT3, CCL5, SCIN, KCNMA1, ITM2C, TP53, MYC, CLU, ZC3H12A, ADIPOQ, CCL2, S100A9, B4GALT1*	24
biological process	GO:0043065	positive regulation of apoptotic process	< 0.001	*PYCARD, MMP9, PPARG, MEF2C, TNF, MSX2, FAP, F2R, CAV1, CCL5, SCIN, KCNMA1, ITM2C, TP53, MYC, CLU, ZC3H12A, ADIPOQ, CCL2, S100A9, B4GALT1*	21
biological process	GO:0043068	positive regulation of programmed cell death	< 0.001	*PYCARD, MMP9, PPARG, MEF2C, TNF, MSX2, FAP, F2R, CAV1, CCL5, SCIN, KCNMA1, ITM2C, TP53, MYC, CLU, ZC3H12A, ADIPOQ, CCL2, S100A9, B4GALT1*	21
biological process	GO:0010941	regulation of cell death	< 0.001	*PYCARD, NOS3, MMP9, MAPT, CORO1A, KITLG, TEK, PPARG, MEF2C, TNF, MSX2, CXCL10, FAP, ANGPTL4, RSL1D1, F2R, CAV1, PIM1, C1QA, MT3, CCL5, SCIN, KCNMA1, ITM2C, TP53, GPR37L1, MYC, CLU, ZC3H12A, ADIPOQ, CITED2, CCL2, FHL2, S100A9, B4GALT1, LTF*	36

biological process	GO:0042981	regulation of apoptotic process	< 0.001	*PYCARD, NOS3, MMP9, CORO1A, KITLG, TEK, PPARG, MEF2C, TNF, MSX2, CXCL10, FAP, ANGPTL4, RSL1D1, F2R, CAV1, PIM1, MT3, CCL5, SCIN, KCNMA1, ITM2C, TP53, MYC, CLU, ZC3H12A, ADIPOQ, CITED2, CCL2, FHL2, S100A9, B4GALT1, LTF*	33
biological process	GO:0043067	regulation of programmed cell death	< 0.001	*PYCARD, NOS3, MMP9, CORO1A, KITLG, TEK, PPARG, MEF2C, TNF, MSX2, CXCL10, FAP, ANGPTL4, RSL1D1, F2R, CAV1, PIM1, MT3, CCL5, SCIN, KCNMA1, ITM2C, TP53, MYC, CLU, ZC3H12A, ADIPOQ, CITED2, CCL2, FHL2, S100A9, B4GALT1, LTF*	33
biological process	GO:0008219	cell death	< 0.001	*PYCARD, NOS3, MMP9, MAPT, CORO1A, KITLG, TEK, CDCA7, CHI3L1, PPARG, DIO3, MEF2C, TNF, MSX2, CXCL10, SHISA5, FAP, ANGPTL4, RSL1D1, F2R, CAV1, PIM1, C1QA, MT3, CCL5, SCIN, KCNMA1, PMP22, ITM2C, TP53, GPR37L1, MYC, CLU, ZC3H12A, ADIPOQ, CITED2, CCL2, CIDEC, FHL2, S100A9, B4GALT1, LTF*	42
biological process	GO:0006915	apoptotic process	0.001	*PYCARD, NOS3, MMP9, CORO1A, KITLG, TEK, CDCA7, CHI3L1, PPARG, DIO3, MEF2C, TNF, MSX2, CXCL10, SHISA5, FAP, ANGPTL4, RSL1D1, F2R, CAV1, PIM1, MT3, CCL5, SCIN, KCNMA1, ITM2C, TP53, MYC, CLU, ZC3H12A, ADIPOQ, CITED2, CCL2, CIDEC, FHL2, S100A9, B4GALT1, LTF*	38
biological process	GO:0012501	programmed cell death	0.002	*PYCARD, NOS3, MMP9, CORO1A, KITLG, TEK, CDCA7, CHI3L1, PPARG, DIO3, MEF2C, TNF, MSX2, CXCL10, SHISA5, FAP, ANGPTL4, RSL1D1, F2R, CAV1, PIM1, MT3, CCL5, SCIN, KCNMA1, ITM2C, TP53, MYC, CLU, ZC3H12A, ADIPOQ, CITED2, CCL2, CIDEC, FHL2, S100A9, B4GALT1, LTF*	38
biological process	GO:0008285	negative regulation of cell proliferation	0.002	*CAV2, PPARG, TNF, MSX2, FAP, DDAH1, F2R, NFIB, SCIN, CNN1, TP53, ADIPOQ, CCL2, B4GALT1*	14
biological process	GO:1904035	regulation of epithelial cell apoptotic process	0.005	*TEK, TNF, ANGPTL4, CCL2*	4
